# SEN1990 is a predicted winged helix-turn-helix protein involved in the pathogenicity of *Salmonella enterica* serovar Enteritidis and the expression of the gene *oafB* in the SPI-17

**DOI:** 10.3389/fmicb.2023.1236458

**Published:** 2023-11-03

**Authors:** Guillermo Hoppe-Elsholz, Alejandro Piña-Iturbe, Omar P. Vallejos, Isidora D. Suazo, Javiera Sepúlveda-Alfaro, Patricia Pereira-Sánchez, Yohana Martínez-Balboa, Eduardo A. Catalán, Pablo Reyes, Valentina Scaff, Franco Bassi, Sofia Campos-Gajardo, Andrea Avilés, Carlos A. Santiviago, Alexis M. Kalergis, Susan M. Bueno

**Affiliations:** ^1^Millennium Institute on Immunology and Immunotherapy, Facultad de Ciencias Biológicas, Pontificia Universidad Católica de Chile, Santiago, Chile; ^2^Departamento de Bioquímica y Biología Molecular, Facultad de Ciencias Químicas y Farmacéuticas, Universidad de Chile, Santiago, Chile; ^3^Departamento de Endocrinología, Facultad de Medicina, Escuela de Medicina, Pontificia Universidad Católica de Chile, Santiago, Chile

**Keywords:** *Salmonella enterica* ser. Enteritidis, pathogenicity island, excisable genomic island, ROD21, SPI-17, gene regulation, *oafB*, *SEN1990*

## Abstract

Excisable genomic islands (EGIs) are horizontally acquired genetic elements that harbor an array of genes with diverse functions. ROD21 is an EGI found integrated in the chromosome of *Salmonella enterica* serovar Enteritidis (*Salmonella* ser. Enteritidis). While this island is known to be involved in the capacity of *Salmonella* ser. Enteritidis to cross the epithelial barrier and colonize sterile organs, the role of most ROD21 genes remains unknown, and thus, the identification of their function is fundamental to understanding the impact of this EGI on bacterium pathogenicity. Therefore, in this study, we used a bioinformatical approach to evaluate the function of ROD21-encoded genes and delve into the characterization of *SEN1990*, a gene encoding a putative DNA-binding protein. We characterized the predicted structure of SEN1990, finding that this protein contains a three-stranded winged helix-turn-helix (wHTH) DNA-binding domain. Additionally, we identified homologs of SEN1990 among other members of the EARL EGIs. Furthermore, we deleted *SEN1990* in *Salmonella* ser. Enteritidis, finding no differences in the replication or maintenance of the excised ROD21, contrary to what the previous Refseq annotation of the protein suggests. High-throughput RNA sequencing was carried out to evaluate the effect of the absence of *SEN1990* on the bacterium’s global transcription. We found a downregulated expression of *oafB*, an SPI-17-encoded acetyltransferase involved in O-antigen modification, which was restored when the deletion mutant was complemented ectopically. Additionally, we found that strains lacking *SEN1990* had a reduced capacity to colonize sterile organs in mice. Our findings suggest that *SEN1990* encodes a wHTH domain-containing protein that modulates the transcription of *oafB* from the SPI-17, implying a crosstalk between these pathogenicity islands and a possible new role of ROD21 in the pathogenesis of *Salmonella* ser. Enteritidis.

## Introduction

*Salmonella enterica* subsp. *enterica* (*Salmonella enterica*) is one of the main causes of foodborne diseases of bacterial origin worldwide (CDC National Enteric Disease Surveillance, 2018; European Centre for Disease Prevention and Control, 2022). Consumption of contaminated food products with non-typhoidal salmonellae serovars, such as Enteritidis, Typhimurium, or Infantis, commonly causes non-typhoidal salmonellosis (NTS) characterized by a local self-limited infection of the intestine (Schultz et al., 2021). Although infections with non-typhoidal salmonellae are normally resolved without complications in immunocompetent individuals, a 2010 estimation reported that NTS accounted for approximately 93 million enteric infections and 155,000 associated deaths worldwide, making it a considerable worldwide burden (Majowicz et al., 2010). Moreover, in children, the elderly, and immunocompromised populations, oral exposure to non-typhoidal salmonellae can result in cases of invasive NTS (iNTS), which are characterized by the invasion of the bacterium beyond the intestines and the colonization of sterile organs. This type of infection has higher lethality than NTS, causing 3.4 million infections and 681,316 deaths annually (Balasubramanian et al., 2018).

The great success of *Salmonella enterica* as a human and animal pathogen may be due to the high plasticity of its genome, which has allowed this species to acquire several virulence factors, as well as host colonization and adaptation genes (Laing et al., 2017). A common source of new genes is through the horizontal acquisition of genetic elements, such as a class of mobile elements known as excisable genomic islands (EGIs). Similar to other genomic islands, EGIs are DNA regions that harbor a wide array of genes with functions related to virulence, metabolism, antimicrobial/heavy metal resistance, and niche colonization (Juhas et al., 2009). They can be recognized from the host chromosome as they differ in sequence signatures, such as GC content, tetranucleotide frequency, and codon usage bias (Juhas et al., 2009). Unlike other genomic islands, EGIs have the capacity to excise from their host chromosome as circular elements through site-specific recombination and are frequently found integrated in *tRNA* or *tmRNA* genes flanked by short direct repeated sequences (DRS) at both ends of the island: *attL* (attachment site Left) and *attR* (attachment site Right; Nieto et al., 2016). The DRS are substrates for site-specific recombination, which is driven by proteins encoded within the island, the host chromosome, or in other mobile elements, resulting in the excision of the island and the generation of two identical sequences—one in the chromosome (*attB*) and the other in the circular island (*attP*). Then, these sequences act as substrates for a subsequent reverse reaction that leads to the integration of the EGI. Additionally, other proteins encoded by the EGI may aid the site-specific recombination reaction, such as the recombination directionality factor (RDF), which drives the reaction in favor of the island excision (Piña-Iturbe et al., 2022). Although the excision of EGIs has been well documented, little is known about their extrachromosomal stage. In this form, EGIs may be transferred to other hosts through phage transduction or conjugation, and as seen in some EGIs, they might replicate through rolling-circle replication like plasmids (Lee et al., 2010; Guérillot et al., 2013), which was previously thought to occur only by replication of their host chromosomes while they were integrated (Burrus and Waldor, 2004). Furthermore, some islands have been reported to decrease the expression of their harbored genes (Godfrey et al., 2011), while others have shown the opposite effect (Tobar et al., 2013), leading to the speculation that excision might be a form of gene regulation (Nieto et al., 2016). Therefore, the excision of EGIs and their maintenance as extrachromosomal elements could be a highly regulated process required for their correct function and lifecycle.

The region of difference 21 (ROD21) is a *Salmonella enterica* EGI identified by Thomson et. al. (2008) as a genomic island present in serovar Enteritidis and Dublin but absent from serovar Typhimurium. This EGI is 27 kb long and belongs to a family of EGIs found integrated into the chromosome of animal and plant pathogens from different families of the order Enterobacterales (Piña-Iturbe et al., 2018). ROD21 contains 28 ORFs, most of which have an unknown function, and only a few have had their function evaluated—a tyrosine-recombinase (Tobar et al., 2013), a Toll-like domain-containing protein (Xiong et al., 2019), an RDF (Piña-Iturbe et al., 2022), and an H-NS-like protein (Fitzgerald et al., 2020). Even so, the lack of knowledge on the function of most genes carried by ROD21 is concerning due to the involvement of the island in the bacterium pathogenicity. Previous studies have shown that strains lacking ROD21, or strains with impaired ROD21 that cannot excise, are less virulent than wild-type (WT) isogenic strains (Tobar et al., 2013). This reduction in virulence is specifically due to the reduced ability of serovar Enteritidis to cross the intestinal epithelium barrier (Pardo-Roa et al., 2019). Additionally, the rate of excision of ROD21 has been reported to be affected by conditions related to the intra-macrophage milieu, such as low pH and nutrient-deprived media (Quiroz et al., 2011). Previous data suggest that ROD21 encodes unidentified virulence factors, improving the capacity of the bacterium to traverse the intestinal epithelium and machinery to sense and respond to immunological stimuli. Therefore, further characterization of the gene content of ROD21 could reveal more information on the mechanisms of pathogenicity of *Salmonella* ser. Enteritidis and possibly other pathogens that carry similar EGIs. Thus, in this study, we have used an *in silico* approach to identify the possible function of the genes encoded by ROD21. We focused on the characterization of a putative DNA-binding protein, which is encoded by the gene *SEN1990*. We then conducted bioinformatic and functional analyses of the protein through *in vivo* studies in a murine infection model to determine its involvement in the pathogenicity of *Salmonella* ser. Enteritidis.

## Methods

### Bioinformatic analyses

The *Salmonella* ser. Enteritidis strain P125109 genome (NC_011294) was downloaded from the Genbank and visualized using SnapGene software (v6.2.1). Primers and probes were designed using Primer Express™ Software.

The SEN1990 DNA-binding domain was identified using the online tools CDD-NCBI (Lu et al., 2019) by searching the amino acid sequence of SEN1990 (WP_001095779.1). Protein modeling and functional characterization through structure homology were performed using the I-Tasser or D-I-Tasser online server (Yang and Zhang, 2015) and Phyre2 (Kelley et al., 2015) with default settings. The amino acid sequences of the RepL proteins from plasmids pMO1 (NC_002059), pSN2 (NC_005565), pNE131 (NC_001390), pIM13 (NC_001376), and pE5 (M17990.1) were obtained from the original publications for the RepL annotation (Lampson and Parisi, 1986; Hefford et al., 1997). The MarR protein structure was downloaded from the PDB database (Alekshun et al., 2001). Model rendering and structural analysis were performed and processed in Pymol2 (PyMOL v1.2r3pre). Structural alignment was performed using the ColorByRMSD script, and the electrostatic map was calculated through ABPS Electrostatics using the psb2pqr method. The isoelectric point and molecular weight of SEN1990 were calculated in the online server https://web.expasy.org/compute_pi/.

SEN1990 homologs among Enterobacteriaceae-associated ROD21-like (EARL) GIs were identified using tBLASTx through the BLAST web server and Easyfig v2.2.5. The protein alignments were performed through MUSCLE multiple amino acid sequence alignment using MEGA11 software with the standard configuration. The multiple alignment figures were rendered through the ESPript 3.0 web server using the “%MultAlin” sequence similarity depiction. A phylogenic tree was then calculated using the IQTREE webserver (Trifinopoulos et al., 2016) with 1,000 bootstraps. The JTTDCMut+G4 substitution model was chosen according to the Bayesian Information Criterion calculated by ModelFinder (Kalyaanamoorthy et al., 2017). The tree was rendered using the iTOL webserver (Letunic and Bork, 2021).

### Bacterial strains and plasmids

The expression plasmid pAPI-*SEN1990* was constructed using the low copy number plasmid pET-15b as a backbone, which was linearized through PCR using the primers Lineal_pET-Fw and Lineal_pET-Rv (Supplementary Table 1). Then, using the high-fidelity polymerase Platinum SuperFi II (Invitrogen 12,368,010), the IPTG-inducible promoter Trc was amplified from pTrc-HisB, adding overhangs homologous to pET15b upstream to the promoter and to *SEN1990* downstream to the promoter. In the same manner, *SEN1990* was amplified by adding overhangs homologous to pET15b downstream of the gene and to ptrC upstream of the gene. The fragments were assembled through Gibson Assembly with the NEBuilder HiFi DNA Assembly buffer (NEB E2621). The fragments were mixed in a proportion of 2:3 (fragment: vector), and the reaction was performed at 50°C for 15 min.

Two *Salmonella* ser. Enteritidis strains were constructed—a deletion mutant lacking *SEN1990* (*Salmonella* ser. Enteritidis ∆*SEN1990::frt*) and a deletion mutant with an inducible plasmid expressing *SEN1990* ectopically (*Salmonella* ser. Enteritidis ∆*SEN1990::frt* pAPI-*SEN1990*).

*Salmonella* ser. Enteritidis ∆*SEN1990::fer.* was constructed through the Lambda Red system (Datsenko and Wanner, 2000) using *Salmonella* ser. Enteritidis PT4 P125109 as the parental strain. Before the elimination of the resistance cassette with plasmid pCP20 (Datsenko and Wanner, 2000), the mutation on the genome of *Salmonella* ser. Enteritidis ∆*SEN1990::cat* was transferred to a clean genetic background through general transduction using P22 phage. The absence of lysogens and pseudolysogens was verified by seeding the bacterium in Evans Blue agar and assessing its susceptibility to infection by the P22 H5 lytic phage.

The complemented strain was constructed by electroporating *Salmonella* ser. Enteritidis ∆*SEN1990::frt* with 200 ng of the plasmid pAPI-*SEN1990*. After 3 h of recovery at 37°C in LB, the culture was seeded in LB agar with 100 μg/ml ampicillin and incubated at 37°C for further selection.

GFP-expressing strains were constructed by electroporation of the pKK233.2-*gfp* plasmid as described above.

All strains constructed were confirmed by PCR (Supplementary Figures 1, 2). Strains were stored in BHI-Glycerol 20% (v/v) at −80°C and cultured in LB broth at 37°C with agitation and with 10 μg/ml chloramphenicol or 100 μg/ml ampicillin when needed.

### PCR and qPCR protocols

DNA amplification by PCR was carried out using either the GoTaq G2 Flexi DNA polymerase (Promega M7801) or the Platinum SuperFi II high-fidelity polymerase. The thermocycler protocols were designed according to the polymerase manufacturers using the primers listed in Supplementary Table 1.

Quantitative PCR (qPCR) amplifications were carried out using either the TaqMan™ Fast Advanced Mastermix (Applied Biosystems 4,444,557) or the qPCR SsoAdvanced Universal SYBR green Supermix (Bio-Rad MH5H1EE8Z) in a StepOne Plus real-time thermocycler, using the protocols provided by their respective manufacturers and the primers listed in Supplementary Table 2.

### Gene expression and ROD21 copy number

Gene expression, island excision, and ROD21 relative copy number assays were carried out by diluting overnight (O/N) cultures of the *Salmonella* ser. Enteritidis WT and the the Δ*SEN1990::frt* mutant strain in 15 ml of LB broth with 0.1 mM L-arabinose to OD_600_ = 0.01 and incubating for 2.5 h at 37°C with shaking, after which samples for DNA and RNA isolation were obtained. RNA was isolated using TRIzol (Invitrogen 15,596,026) according to the manufacturer’s protocol. Residual DNA was removed with the Turbo DNA-Free kit (Invitrogen AM1907), and cDNA was synthesized with the iScript cDNA synthesis kit (Bio-Rad M87EWZESH). The mRNA quantity of *oafB* was quantified using SsoAdvanced Universal SYBR Green Supermix, and the relative gene expression was calculated with the 2^-(∆∆Ct)^ equation, using *rpoD* as the endogenous control and the WT strain as the reference. For the island copy number and excision assays, genomic DNA was extracted using phenol:chloroform:isoamyl alcohol as previously described (Piña-Iturbe et al., 2022). The qPCR amplifications were carried out using TaqMan™ Fast Advanced Master Mix. The copy number of *attB* and *rpoD* in each DNA sample was calculated by interpolating the Ct of both amplicons into their corresponding standard curve made of tenth-fold dilutions of *Salmonella* Typhimurium genomic DNA (10^8^–10^2^ total chromosomes), as it naturally lacks ROD21. The copy number of *SEN1998* was calculated using a standard curve of the plasmid pET15b-*SEN1998* (10^8^–10^2^ total plasmid copies). Island excision was calculated by the relation: % Excision = 100 × *attB*/*rpoD* in each sample. The relative copy number of ROD21 was calculated by the ratio of *SEN1998* copies/*rpoD* copies. The relative number of episomal ROD21 copies was calculated by normalizing the Ct of *attP* to *rpoD* within a sample and then to the WT strain (control sample) through the 2^-(∆∆Ct)^ equation.

### RNA sequencing

The *Salmonella* ser. Enteritidis WT and Δ*SEN1990::frt* strains were cultured O/N at 37°C in LB broth with agitation and diluted in LB to OD_600_ = 0.01. The cultures were incubated at 37°C with agitation until OD_600_ = 0.6, after which samples were centrifugated at 8,000 *g* for 8 min. The supernatant was discarded, and RNA was isolated using TRizol. The samples were further purified from salts using drop dialysis in MF-Millipore™ membranes (0.22 μm pore size; Millipore GSWP02500) against nuclease-free water for 4 h on ice. RNA-seq was performed by Zymo Research as follows: libraries were prepared using the Zymo-Seq RiboFree Total RNA Library Prep Kit and sequenced on an Illumina NovaSeq platform to a sequencing depth of at least 30 million read pairs (150 bp paired-end sequencing) per sample. The bioinformatic analysis was performed as follows: quality control of raw reads was carried out using FastQC v0.11.9. Adapters and low-quality sequences were trimmed from raw reads using Trim Galore! v0.6.6. and the trimmed reads were aligned to the reference genome using STAR v2.6.1d. BAM file filtering, and indexed using SAMtools. RNAseq library quality control was implemented using RSeQC v4.0.0 and QualiMap v2.2.2-dev. Duplicate reads were marked using Picard tools v2.23.9. Library complexity was estimated using Preseq v2.0.3. Duplication rate quality control was performed using dupRadar v1.18.0. Reads overlapping with coding sequences were assigned to genes using featureCounts v2.0.1. Classification of rRNA genes and their reads was based on annotations and RepeatMasker rRNA tracks from the UCSC genome browser when applicable. Differential gene transcription analysis was completed using DESeq2 v1.28.0. Functional enrichment analysis was achieved using g:Profiler python API v1.0.0. Quality control and analysis results plots were visualized using MultiQC v1.9. BAM indexing and read coverage plots were performed with IGV 2.16.0.

### LPS extraction and analyses

The LPS samples were prepared as previously described (Marolda et al., 2006), with some modifications. Briefly, bacteria were grown in LB at 37°C with agitation (160 rpm) until reaching an OD_600_ = 0.6. An aliquot of each culture was adjusted to an OD_600_ = 2.0 with PBS. Next, 1.5 ml of each suspension was centrifuged, and bacterial pellets were suspended in 90 μl of lysis buffer (1 M Tris–HCl pH 6.8, 2% SDS, 4% β-mercaptoethanol, 10% glycerol, and 0.002% bromophenol blue) and incubated for 10 min in a boiling water bath. Finally, each lysate was supplemented with 10 μl of proteinase K (10 mg/ml) and incubated for 1 h at 60°C. The LPS samples (5 μl) were analyzed in 12% (w/v) acrylamide gels using a Tricine-SDS buffer system (Lesse et al., 1990) and visualized by silver staining (Marolda et al., 2006).

### Mouse infection assays

For the mouse survival assays, bacterial doses were prepared by diluting O/N cultures to an initial OD_600_ = 0.01 in 3 ml of LB broth and incubated at 37°C and 160 rpm until OD_600_ = 0.6 was reached. Growth was halted by briefly placing the culture tubes in ice. Then, the exact volume of the culture required for a dose of 1 × 10^6^ CFU (colony forming unit) in 50 μl was diluted in sterile PBS. This volume was calculated considering 3 × 10^8^ CFU/μl in OD_600_ = 0.5, as estimated from previous CFU counts. Then, 6–7 weeks old male C57BL/6 mice were administered intragastrically with 50 μl of SEn WT, SEn Δ*SEN1990::frt*, SEn-*aph*, SEn Δ*SEN1990::cat*, or PBS. The mice were monitored throughout the assay while measuring their daily weight and clinical score from 0 to 15, with 0 being a normal state and 15 the state of maximum distress and suffering. A clinical score equal to 8 or a loss of more than 20% of the initial weight was used as a threshold to apply euthanasia (Sebastián et al., 2022). The clinical parameters evaluated are described in Supplementary Table 3.

For bacterial competition assays, bacterial doses were prepared as described above. Mice were infected intragastrically with 50 μl of a mixture of 5 × 10^5^ CFU of SEn WT-*aph* and 5 × 10^5^ CFU of SEn Δ*SEN1990::cat*. The mice were monitored throughout the assay while measuring their daily weight and clinical score. Forty-eight hours after the initial inoculum, the mice were euthanized, and complete organs were extracted, namely blood, liver, spleen, mesenteric lymph node, gallbladder, colon, and ileum. The organ samples were homogenized in sterile PBS, and serial dilutions were carried out. The bacterial load was assessed by culturing the serial dilutions on LB and *Salmonella-Shigella* agar plates with 50 ng/μl kanamycin or 10 ng/μl chloramphenicol. The CFUs were then counted after 24 h of incubation at 37°C. The *Salmonella-Shigella* agar was used to confirm that the colonies found in the sterile organs were *Salmonella*. The competition index was calculated as the ratio of the bacterial load recovered from LB-chloramphenicol plates by the bacterial load recovered from the LB-kanamycin plates (i.e., Δ*SEN1990::cat*/WT-*aph*) and normalized with the ratio of the input inoculum to account for differences in the initial CFUs of each strain in the mixture.

To evaluate the infection of the strains individually, the doses were prepared as described above, and mice were inoculated intragastrically with 50 μl of 1 × 10^6^ CFUs SEn WT-*aph* or 1 × 10^6^ CFU of SEn Δ*SEN1990::cat*. After 48 h post-infection (hpi), the mice were euthanized, and the organs were collected and processed as described above. The bacterial load was reported as CFU per gram of organ or ml of blood. The same assay was performed using the strains without antibiotic resistance genes, SEn WT, or SEn Δ*SEN1990::frt,* but the samples were seeded on LB and *Salmonella-Shigella* agar plates without antibiotics.

For all the analyses, we defined a limit of detection (LOD) as counting a minimum of one CFU in the 30 μl LB agar seeded of each organ sample. Thus, when no CFUs were detected, the LOD was applied.

### Bacterial uptake by murine RAW264.7 macrophages

RAW264.7 cells were incubated at 37°C and CO_2_ 5% in DMEM medium supplemented with 10% FBS until confluence. Then, cells were scraped in fresh medium and diluted to a final concentration of 5 × 10^5^ cells/ml. Each well in a 24-well plate was filled with 1 ml of the suspension of cells. The bacterial doses were prepared by inoculating 3 ml of LB medium with O/N-grown *Salmonella* ser. Enteritidis WT pKK233.2-*gfp* and ∆*SEN1990::frt* pKK233.2-*gfp* to an initial OD_600_ = 0.15 and grown at 37°C and shaken until OD_600_ = 0.6. After this, the RAW264.7 cells were infected at an MOI = 10 bacteria/cell. To ensure infection, the plates were centrifuged at 2,000 *g* for 3 min after bacterial inoculation and before incubation at 37°C with 5% CO_2_. After 2 h of infection, 10 μl of gentamicin (10 μg/ml) was added to the cells, which were further incubated for 1 h at 37°C with 5% CO_2_, after which they were centrifuged at 2,000 *g* for 5 min. The media were replaced with 250 μl of PBS, and the cells were scraped and transferred to a 96-well plate where they were centrifuged and washed again with PBS. Then, the cells were stained with 50 μl of the viability probe (Alexa Fluor 700) for 10 min at 4°C, washed in PBS once, and fixed in 4% paraformaldehyde for 15 min at 4°C. The cells were then washed once with PBS and resuspended in 200 μl of PBS. The samples were stored O/N and analyzed the next day using the flow cytometer BD LSRFortessa™ X-20 with the software BD FACSDiva™. Using control samples, the voltage settings and compensation were adjusted before the main data acquisition. The singlets gating was performed by selecting the events that followed a 1:1 relationship between SSC-A and SSC-H. Then, RAW264.7 cells were gated by their size and complexity detected by the FSC-A and SSC-A, respectively. The filters FITC and Alexa-700A were used for GFP and viability probe detection and gating. Data processing and the gating were carried out using FlowJo V10.

### Statistical analyses

Statistical analyses were performed using GraphPad Prism 10 software. All statistical analyses were performed with α = 0.05, and the error bars on the graphs represented the standard deviation.

The gene expression, relative ROD21 copy number, and island excision statistics were analyzed through a one-way ANOVA or *t*-test for independent samples. RNA-seq differential gene transcription statistics were assessed by calculating the false discovery rate (FDR) of each gene with a foldchange threshold = 0 and setting the significance as FDR < 0.05.

Competitive indexes were log-transformed and analyzed through one sample *t*-test with a theoretical mean of 0, which implied that when no statistical differences are detected, the bacterial load of both strains is equal. Bacterial loads in independent infections were analyzed with a *t*-test for independent samples. Mouse weight and clinical score were analyzed through a two-way ANOVA with time post-infection and strain as factors. Mouse survival curves were compared using the log-rank (Mantel-Cox) test.

### Ethics approval statement

The animal work was reviewed and approved by the Institution Scientific Ethical Committee for Animal and Environmental Care from Pontificia Universidad Católica de Chile under the approval code: 210707009.

## Results

### Structural homology and domain analyses reveal new information on the function of ROD21-encoded hypothetical proteins

Within ROD21, there are 28 ORFs, of which there are only experimental data regarding the function of *SEN1970* (Integrase), *SEN1993* (H-NS-like), *SEN1975* (TcpS), and *SEN1998* (RDF). For the remaining 24 ORFs, information regarding their putative function in the RefSeq annotation (NCBI Prokaryotic Genome Annotation Pipeline (PGAP)) is only available for some genes; *SEN1976*, *SEN1977*, and *SEN1978* encode type IV pili-related proteins (pilus assembly protein PilV-like, phage tail fiber protein, and type IV major pilin), *SEN1979* encodes a TradD-like protein, *SEN1980* encodes a MobQ family relaxase, *SEN1982* and *SEN1984* encode lipoproteins, *SEN1985* encodes a putative S-adenosyl-L-methionine dependent methyltransferase, *SEN1990* encodes a putative plasmid replication/maintenance protein, and *SEN1994* and *SEN1995* encode a putative conflict system composed of a SLATT domain-containing protein and a nucleotidyltransferase, respectively (Table 1).

**Table 1:** ROD21 uncharacterized proteins with pBLAST (a) or Phyre2 (b) results.

Protein	pBlast^a^	Phyre2^b^
SEN1977	Tail fiber protein (*Salmonella*) ID: 100%, Cov 100% WP_001127942.1	Long tail fiber protein p37 Conf: 100%; ID: 34%; Cov: 24% PDB: 2XGF
SEN1978	Type 4 pilus major pilin (*Enterobacteriaceae*) ID: 100%, Cov: 100% WP_000243508.1	Type IVb pilin (PilS) from *Salmonella* Typhi Conf: 99.5%; ID: 23%; Cov: 76% PDB: 1Q5F
SEN1979	Conjugal transfer protein TraD (*Salmonella*) ID: 100%, Cov: 100% WP_000368357.1	-
SEN1980	MobQ family relaxase (*Salmonella*) ID: 100%, Cov: 100% WP_000964102.1	Nicking enzyme Conf: 100%; ID: 30%; Cov: 38% PDB: 4HT4
SEN1981a	Membrane protein (*Salmonella enterica* serovar Dublin) ID: 100%, Cov: 100% EGE30161.1	-
SEN1982	Lipoprotein (*Salmonella*) ID: 100%, Cov: 100% WP_000722542.1	PadR-family transcriptional regulator Rv3488 Conf: 90.1%; ID: 23%; Cov: 24% PDB: 5ZHC
SEN1984	Lipoprotein (*Escherichia coli*) ID: 93.6%, Cov: 100% WP_171885974.1	BamE component of the β-barrel assembly Conf: 99.7%; ID: 29%; Cov: 30% PDB: 7TXX
SEN1990	Replication/Maintenance protein RepL (*Salmonella*) ID: 100%, Cov: 100% WP_001095779.1	LysR family transcriptional regulator Conf: 91.5%; ID: 24%; Cov: 45% PDB: 5Y9S
SEN1994	SLATT domain-containing protein (*Salmonella enterica*) ID: 100%, Cov: 100%, E value: 3e-130 WP_231200135.1	-
SEN1995	Nucleotidyltransferase (*Salmonella*) ID: 100%, Cov: 100%, E value: 0.0 WP_000102010.1	Cyclic trinucleotide synthase CdnD Conf: 100%; ID: 19%; Cov: 67% PDB: 7D4S

To uncover more evidence regarding the function of the ROD21 genes, we used Phyre2 to find structural homologs of each putative encoded protein (Table 1). Although this analysis failed to find any reliable predicted homolog for most ROD21 hypothetical proteins, we found new data regarding the function of the two putative lipoproteins—SEN1982 (WP_000722542.1) and SEN1984 (WP_000734572.1). Additionally, we found data that supports the predicted function of some proteins encoded in ROD21, such as the type IV pili-related proteins SEN1977 and SEN1978, the putative conjugal transfer protein SEN1980, and the putative nucleotidyltransferase SEN1995. The lipoprotein SEN1982 was predicted to contain a motif structurally similar to the wHTH domain of PadR-family transcriptional regulators (Table 1), specifically within the recognition helix and the wing (Ser-58 to Val-99). This suggested that SEN1982 could possibly bind to DNA, whereas the other lipoprotein SEN1984 was predicted to be structurally similar to a β-barrel assembly complex component, BamE (Pro-1 to Arg-109; Table 1). Finally, SEN1990 (WP_004860215.1), which is annotated in the RefSeq *Salmonella* ser. Enteritidis genome (NC_011294) as a putative replication/maintenance protein from the RepL family, was predicted by Phyre2 to share structural similarity with a LysR-like transcriptional regulator (Table 1). Other hits with a similar confidence score (> 90%) showed structural similarity to other wHTH-containing proteins, such as proteins from the MecR, MarR, and FadR family of transcriptional regulators. This predicted similarity was restricted between Glu-73 to Leu-181 of SEN1990 and the DNA-binding domains of the transcriptional regulators. Further inspection of the predicted domains in SEN1990 through NCBI-CDD showed that the protein contained a putative MarR-like HTH domain between Arg-62 and Leu-121.

### SEN1990 contains a three-stranded wHTH DNA-binding domain

**Figure 1:**
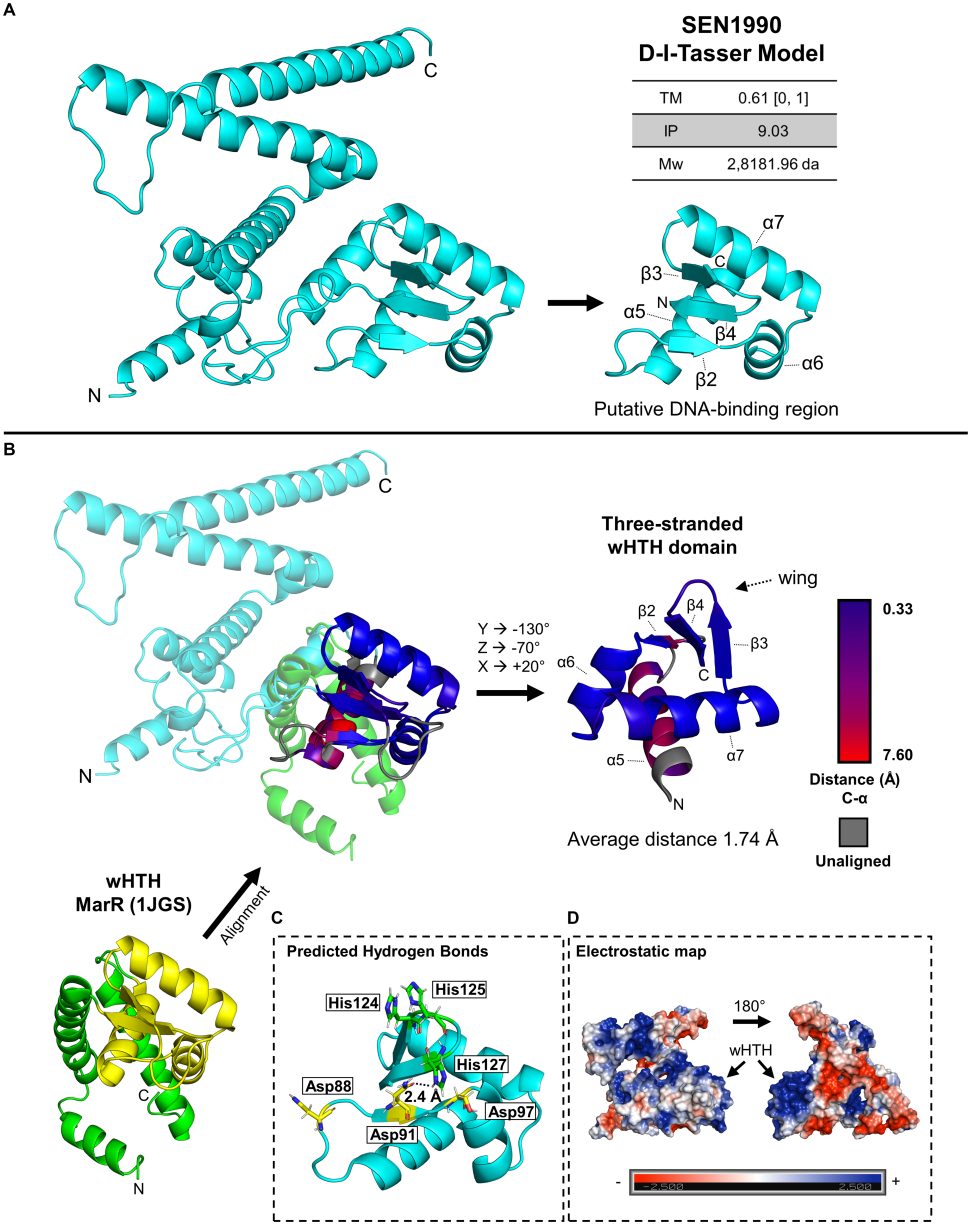
**(A)** Model of SEN1990 calculated by D-I-Tasser. The model TM-score with its scale, the protein isoelectric point (IP), molecular weight (Mw), and the putative DNA-binding region are beside the model. **(B)** Structural alignment of SEN1990 and marR (PDB: 1JGS), a common wHTH domain-containing protein. The wHTH domain in MarR is colored yellow. The aligned structures are colored by the C-α distances between the SEN1990 and MarR aligned residues. The predicted DNA-binding region is shown isolated and rotated next to the alignment. **(C)** Predicted hydrogen bonds by Pymol within the DNA-binding region of SEN1990. Other residues capable of hydrogen contact are highlighted in the model. **(D)** Electrostatic map of SEN1990 calculated through Pymol with the APBS electrostatics plugin.

Among the genes with new predicted functions, *SEN1990* caught our attention since it was previously reported to have an effect on the persistence of *Salmonella* ser. Enteritidis in oviduct cells in chickens (Raspoet et al., 2014), and although not statistically significant, in the colonization of internal mouse organs (Silva et al., 2012). Moreover, since this gene is apparently involved in plasmid replication or maintenance, a possible core function of ROD21, its characterization would deepen our knowledge of the biology of the island. Therefore, to initially characterize its function, we gathered structural data for its encoded protein. Since SEN1990 does not have functionally characterized homologs, structural data were scarce. Therefore, we began the *in silico* structural characterization of SEN1990 by modeling the protein using the deep learning software D-I-TASSER (Zheng et al., 2021), which uses the I-TASSER protein threading pipeline to find structural templates in state-of-the-art protein model databases and generate the model (Yang and Zhang, 2015). The model constructed by the software had a template modeling score (TM-score) of 0.61 on a scale of 0 to 1, with 1 being the highest confidence score in the model (Figure 1A). A TM-score higher than 0.5 indicates a model with correct topology. Additionally, the isoelectric point (IP) calculated for SEN1990 was basic (9.03). Between the residues Lys-71 to Ile-130 of the protein, we identified a domain structurally similar to Helix-Turn-Helix (HTH) domains (Figure 1B). This region of SEN1990 is composed of a bundle of three α-helices (α) arranged triangularly (α5, α6, and α7), common for HTH domains, and three β-Strands (β) with two of them (β4 and β5) having antiparallel orientation that formed a “wing” structure, such as in winged HTH (wHTH) domains (Aravind et al., 2005). Following the predicted secondary structure of the protein, the wHTH was arranged as α5 – β3 – α6 – α7 – β4 – β5. The first β-Sheet (β3) is commonly found on a sub-class of wHTH domains known as three-stranded wHTH (Aravind et al., 2005). Moreover, analysis of the predicted hydrogen bond contacts showed putative interactions between the region connecting the α5 and α6 helices with the positively charged loop in the wing, which is also characteristic of three-stranded wHTH domains (Figure 1C; Aravind et al., 2005). Additionally, in the electrostatic map of SEN1990, there was an accumulation of positive charges in the wHTH domain (Figure 1D). These charges seem to be due to conserved lysine residues in α7, which corresponded topologically with the DNA-recognition helix of wHTH domains, and conserved histidine residues in the wing. The alignment of the putative wHTH region of SEN1990 to MarR (1JGS), a common wHTH domain-containing protein, showed an average distance of 1.74 Å between the aligned C-α atoms (Figure 1B). The alignment distances were as low as 0.33 Å in the region from β3 to β5, whereas α5 was dissimilar to the marR topology. The α5 helix had distances that were as high as 7.6 Å, and even some residues that were not aligned. On the other hand, the rest of SEN1990 (i.e., the N and C terminal regions of the protein) did not share structural similarities with other characterized proteins.

### SEN1990 homologs are present on other enterobacteriaceae-associated ROD21-like family of genomic islands

**Figure 2:**
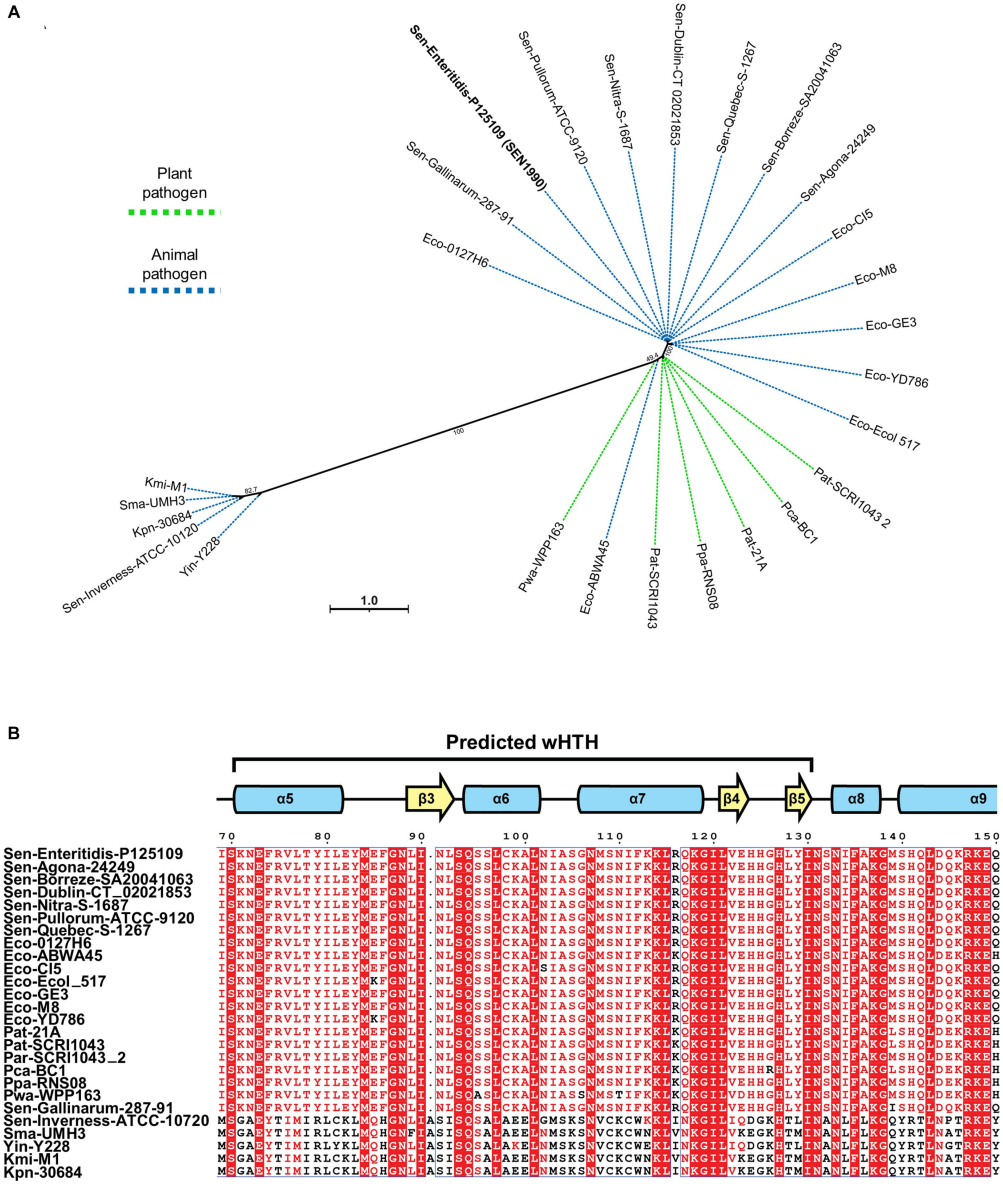
**(A)** Maximum likelihood phylogeny between SEN1990 homologs from EARL EGIs. Green lines indicate EGIs harbored by plant pathogens, and blue lines indicate EGIs harbored by animal pathogens. **(B)** Multiple sequence alignment between the SEN1990 homologs. Only the predicted DNA-binding region is shown. Residues highlighted in red columns are conserved in all proteins, while residues with red letters are highly conserved or share similar biochemical properties. The complete alignment is shown in Supplementary Figure 3.

Since ROD21 belongs to the EARL family of EGIs, and no other characterized proteins shared structural homology with SEN1990, we assessed whether genes similar to *SEN1990* were carried by other EGIs from this family. Through tBLASTx and loci comparison between ROD21 and EARL EGIs, we identified the presence of possible SEN1990 homologs in 25 EGIs harbored by different bacterial strains. Through multiple sequence alignment and phylogenetic analysis, we identified two groups of SEN1990 homologs (Figure 2A). The first one was composed of homologs closely related to SEN1990 harbored by strains of the animal pathogens *E. coli* and *Salmonella* and by the plant pathogen *Pectobacterium*. Moreover, the predicted proteins from this group were highly conserved, as they were almost identical to SEN1990. On the other hand, the second group of homologs was composed of proteins encoded by EGIs carried by strains of the animal pathogens *Klebsiella*, *Yersinia*, *Serratia*, and *Salmonella*. Furthermore, the predicted protein homologs from this group had less amino acid similarity with SEN1990, but they still shared some highly conserved residues, especially within the predicted DNA-binding domain, such as the region between the highly conserved motif between Lys-118 and Leu-121 (Figure 2B). Interestingly, the genetic context of all *SEN1990* homolog loci was similar, as the genes were located in a homologous position within each EGI. Most of the homologous genes have a relatively long intergenic non-coding region followed by a divergently oriented gene upstream from their initial codon, whereas, in the rest of the loci, there are only small predicted ORFs in the intergenic region.

### *SEN1990* is not involved in the replication or maintenance of ROD21

**Figure 3:**
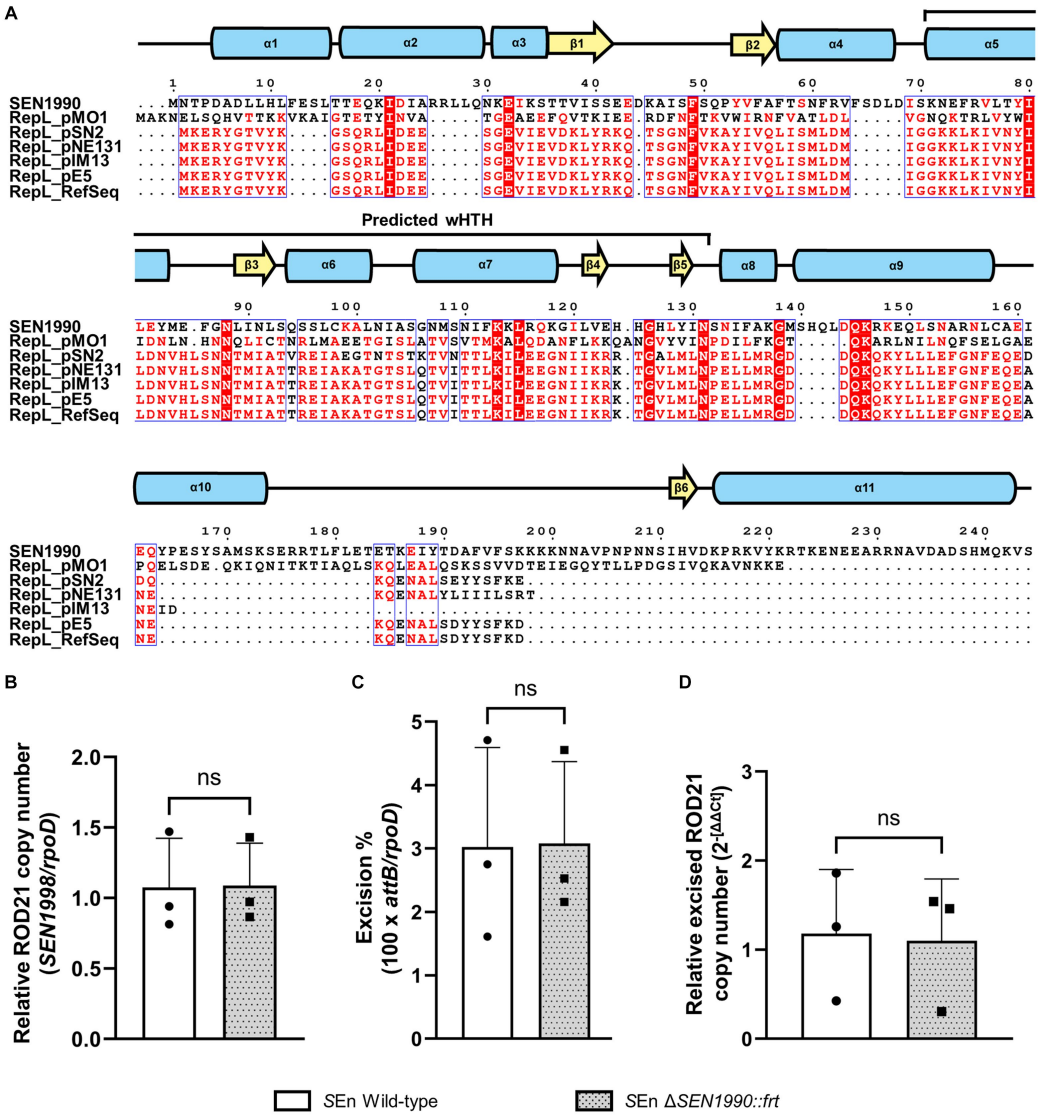
**(A)** Multiple sequence alignment of SEN1990 and the RepL family proteins used in the family evidence. Residues highlighted in red columns are conserved in all proteins, while residues with red letters are highly conserved or share similar biochemical properties. Above the alignment is the representation of the secondary structure of SEN1990 calculated by I-Tasser, and the predicted DNA-binding region by NCBI-CDD is highlighted above the secondary structure. **(B)**. The relative ROD21 copy number in each strain is calculated by the ratio between a ROD21 gene (total ROD21) and *rpoD* (total chromosomes). **(C)** The excision percentage of ROD21 in each strain was calculated as 100 × *attB/rpoD.* **(D)** Relative excised ROD21 between each strain compared to the WT strain calculated through the ΔΔCT method. *t*-test for independent samples α = 0.05. (*p < 0.05).

Since *SEN1990* was annotated through the RefSeq pipeline as encoding a protein from a family of replication/maintenance proteins named RepL, and we found evidence that supports its function as a DNA-binding protein, we sought to assess if *SEN1990* was involved in the replication/maintenance of ROD21. To do this, we first assessed the protein amino acid sequence similarity to other RepL family proteins by multiple sequence alignment between SEN1990 and the plasmid-encoded proteins from the RepL family (Lampson and Parisi, 1986; Hefford et al., 1997). In these results, SEN1990 seemed to be dissimilar to the RepL proteins since there were only a few conserved residues between all proteins (Figure 3A), and the sequence identity fell below 19% between SEN1990 and RepL proteins. Moreover, residues that were highly conserved in RepL proteins, such as Asn-131 and Pro-132, were not present in SEN1990, nor were they substituted by residues with similar properties. This suggested that SEN1990 might not be part of the RepL family or that it was distantly related to these replication/maintenance proteins. Thus, we experimentally assessed whether *SEN1990* was involved in the replication/maintenance of ROD21 despite the protein not sharing strong similarities with RepL proteins. Importantly, the capacity of ROD21 to replicate autonomously and the number of copies in which the excised island can be found within a cell have not been assessed previously. Therefore, we measured the number of copies of ROD21 per chromosome (i.e., total ROD21 copy number) and the relative quantity of excised ROD21. If ROD21 was replicated alongside the chromosome, we could expect an equal amount of ROD21 genes to single copy chromosomal genes, such as *rpoD*, whereas if ROD21 self-replicated, then there would exist more than one copy of ROD21 genes per *rpoD* in the bacterial population. The results showed that the copies of a ROD21 gene, *SEN1998*, were in a ratio of 1 to *rpoD* in the WT strain, suggesting that ROD21 existed as a single copy of EGI per chromosome. Therefore, it was likely that the island was unable to replicate autonomously, and this process probably relied on chromosomal replication (Figure 3B). Nonetheless, the absence of a protein involved in the EGI extrachromosomal maintenance could lower the amount of excised ROD21 to values under the number of total chromosomes, such as in the case of a population of cells losing ROD21 entirely. Moreover, even when *SEN1990* was deleted, we observed that the ratio of ROD21/chromosomes remained 1, indicating that ROD21 was maintained regardless of the presence of *SEN1990*. Despite this, since the excision of ROD21 in all the strains was only 3% (Figure 3C), the replication of a subset of excised ROD21 might not be identifiable among all the copies of ROD21. Therefore, we measured the relative amount of excised ROD21 by amplifying the *attP* sequence, which was only present on the circular ROD21. We observed that in the strain lacking *SEN1990*, the relative quantity of excised ROD21 remained unaltered (Figure 3D), supporting the notion that the protein was not involved in the EGI replication/maintenance.

### SEN1990 modulates the expression of an LPS modification gene from the SPI-17

**Figure 4:**
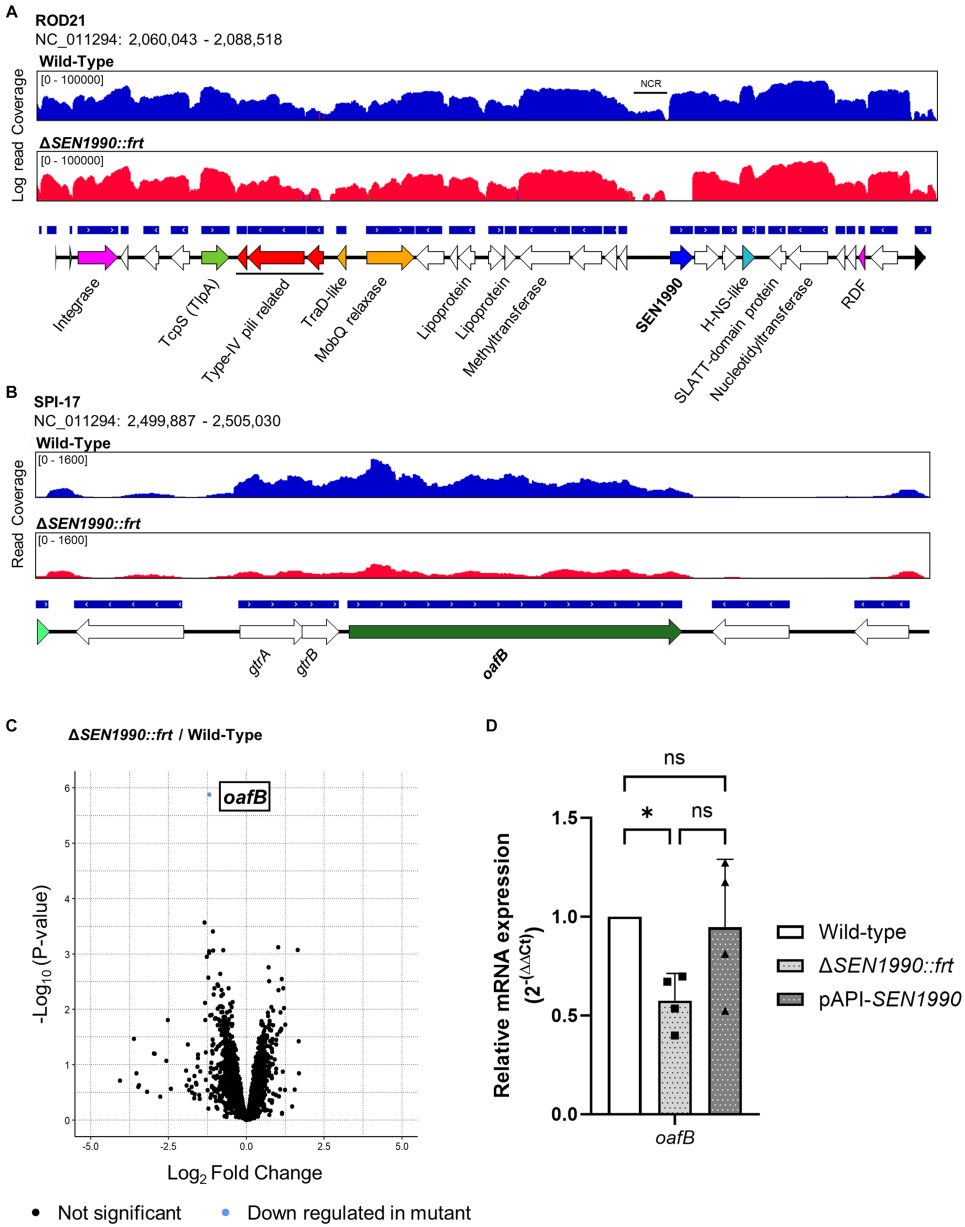
**(A)** RNA-seq read coverage of ROD21 in the WT and Δ*SEN1990::frt* strains. **(B)** RNA-seq read coverage of the SPI-17 island in *Salmonella* ser. Enteritidis WT and Δ*SEN1990::frt* strains. **(C)** Volcano plot of RNA-seq between the WT and Δ*SEN1990::frt* strains. Statistical significance for the differential gene transcription was set to FDR < 0.05. **(D)** Confirmation of the RNA-seq using different samples through RT-qPCR. The relative mRNA quantity of *oafB* between strains was calculated using the 2^-(ΔΔCt)^ method. One-way ANOVA for independent samples α = 0.05. (*p < 0.05).

Since *SEN1990* did not seem to play a role in the replication/maintenance of ROD21 and its predicted product shared structural similarities to wHTH-containing transcriptional regulators, we evaluated if the protein could modulate the transcription of genes in *Salmonella* ser. Enteritidis. Hence, we performed a total RNA-seq of the WT and Δ*SEN1990::frt* and evaluated the differential gene transcription between them. First, deletion of *SEN1990* did not affect the expression of ROD21 genes (Figure 4A), although the gene directly downstream of *SEN1990*, *SEN1991*, had an increased expression in the mutant strain (Log_2_ Fold change = 1.02) but with an FDR of 0.43. Therefore, we assessed through RT-qPCR the transcription of *SEN1991*, which, although not statistically significant, showed that the mRNA quantity of Δ*SEN1990::frt* was higher compared to the WT strain, whereas the complemented strain showed similar expression to the WT strain, hinting at a possible regulation of its own operon (Supplementary Figure 4).

Interestingly, upstream of *SEN1990*, there was an intergenic non-coding region that also had low transcription compared to the rest of the island, hinting at a possible location of the yet unidentified transfer origin of ROD21. Within the *Salmonella* ser. Enteritidis genome, the only gene with a statistically significant differential expression besides *SEN1990* was *oafB* (previously known as Family II *gtrC*), which encodes an LPS modification enzyme located in pathogenicity island SPI-17 (Figure 4A). This gene had a log_2_ fold change of −1.18, meaning that it was approximately expressed 0.44 times less in the mutant compared to the WT strain (Figure 4B). Besides *oafB*, there were several genes downregulated in the mutant strain, although these differences in expression were not statistically significant, and these genes had few reads associated with them. To confirm the RNA-seq result, we performed RT-qPCR of *oafB* in different samples of *Salmonella* ser. Enteritidis grown in the same conditions while also assessing the transcription of the gene in the strain complemented ectopically with *SEN1990* (*Salmonella* ser. Enteritidis Δ*SEN1990::frt* pAPI-*SEN1990*). The expression of *oafB* in Δ*SEN1990::frt* was approximately half of its expression in the WT strain, which supports the results obtained in the RNA-seq analyses. Additionally, the ectopic complementation of *SEN1990* recovered the mRNA quantity of *oafB* near to the levels seen in the WT strain (Figure 4C). Nonetheless, the expression of *oafB* did not reach statistically significant differences between the deletion and complementation strains. To evaluate if the deletion of *SEN1990* could alter the O-antigen of the pathogen, we extracted the LPS from the WT, Δ*SEN1990::frt*, and complemented strains and compared them in a silver-stained SDS-PAGE, but we could not detect any shifts in the LPS bands between the strains (Supplementary Figure 4B).

### *SEN1990* deletion mutants show a reduced capacity to colonize sterile organs in mice

**Figure 5:**
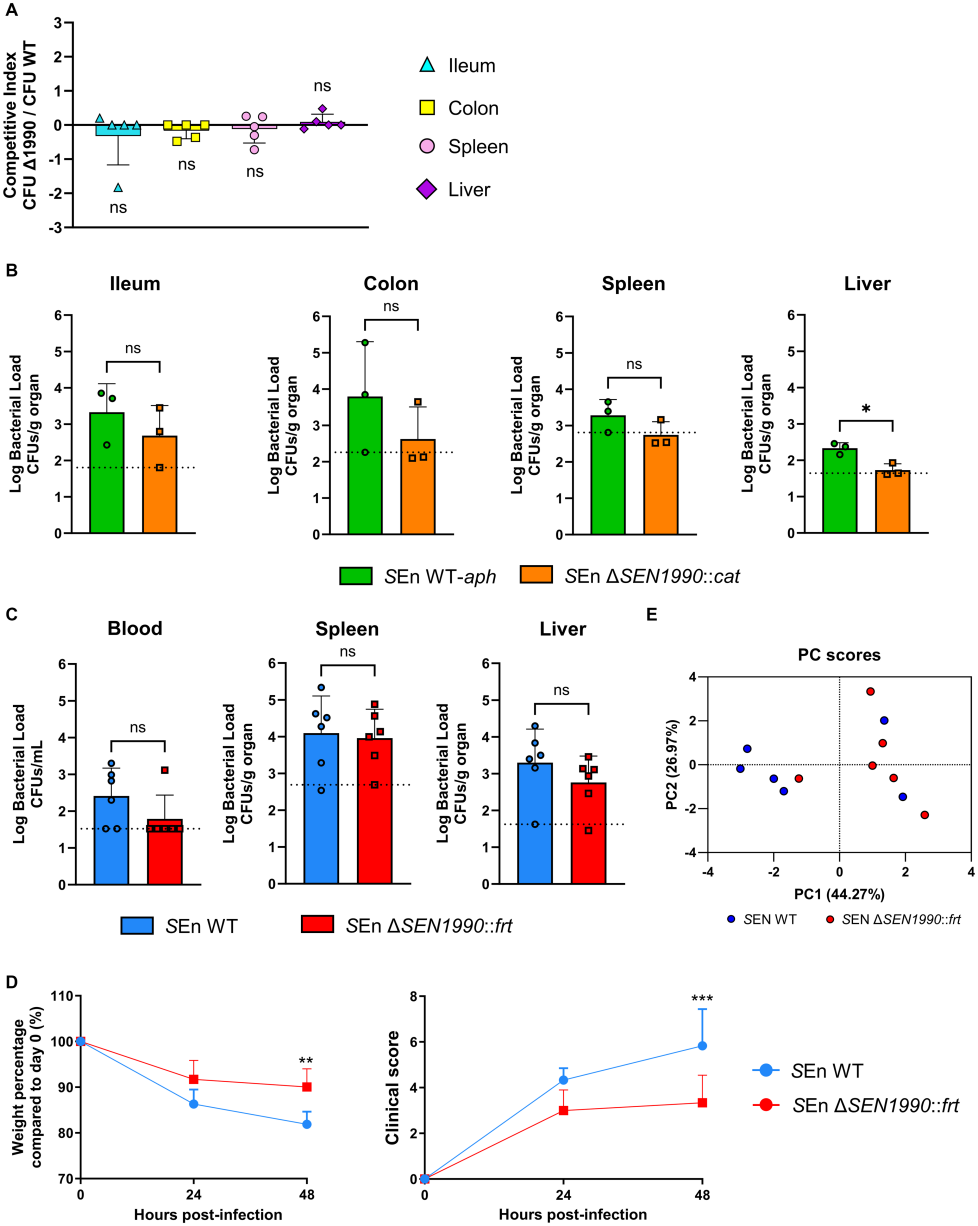
**(A)** Bacterial competition assays. The competition index reflects the ratio between CFUs recovered from SEn Δ*SEN1990::cat* and CFUs recovered from SEn WT-*aph,* normalized against the initial inoculum ratio of the strains. One sample *t*-test α = 0.05. **(B)** Log bacterial CFU recovered from each organ at 48 hpi of the strains marked with an antibiotic resistance gene **(C)** and the strains lacking the resistance marker. The dotted line indicates the LOD for each organ. *t*-test for independent samples α = 0.05 (*p < 0.05). **(D)** Weight and clinical score of the mice throughout the assay. Two-way ANOVA α = 0.05 with Šídák’s multiple comparisons test (**p < 0.01; *** *p* < 0.001). **(E)** PCA of the mice infected with SEn WT or Δ*SEN1990::frt.* The data include weight-loss percentage and clinical score at 48 hpi, log bacterial load in the blood, spleen, liver, mLN, and gallbladder, and length (cm) of colon and ileum. The axis shows the principal components (PC) 1 and 2 and the percentage of variance that they explain in the model.

As stated before, previous studies have shown evidence that *SEN1990* might have a role in the pathogenesis of *Salmonella* ser. Enteritidis through microarray-based screenings (Silva et al., 2012; Raspoet et al., 2014). Moreover, we have shown that *SEN1990* is involved in the expression of another pathogenicity island gene that encodes a protein that catalyzes the modification of LPS. Thus, we hypothesized that *SEN1990* contributes to the pathogenic potential that ROD21 provides to the bacterium. Therefore, to test this hypothesis, we performed an infection competition assay where mice were inoculated intragastrically with a mixture of equal concentrations of *Salmonella* ser. Enteritidis WT strain marked with a kanamycin resistance gene inserted in ROD21 (WT-*aph*) and the Δ*SEN1990::cat* strain. After 48 hpi, there were no differences in the ratio of CFUs recovered of both strains in any of the organs evaluated (Figure 5A). Nonetheless, we also evaluated the pathogenicity of each strain independently since it is possible that a cooperative phenomenon could mask the differences between the strains when they are co-administered. Thus, we inoculated mice intragastrically with either WT-*aph* or Δ*SEN1990::cat*. We observed at 48 hpi that the strain lacking *SEN1990* had a statistically significant reduced bacterial load in the liver while also a lower bacterial load in the ileum, colon, and spleen (Figure 5B). No CFUs of either strain could be recovered from the blood, mesenteric lymph node (mLN), and gallbladder. To rule out a bias on the bacterial loads caused by the insertion of the antibiotic resistance genes, such as a metabolic burden, we performed the experiment using the strains without the selection markers, *Salmonella* ser. Enteritidis WT and Δ*SEN1990::frt*. We observed no statistically significant differences in the bacterial loads between the strains, although there were fewer CFUs of Δ*SEN1990::frt* recovered from the blood (where we did not detect the development of bacteremia in most mice infected with this strain) and the liver (Figure 5C). In contrast to the previous experiment, the bacterial load in the spleen was almost identical in both strains, and CFUs were detected in only a few mice in mLN and gallbladder, but no tendencies could be seen (Supplementary Figure 5). Additionally, mice infected with the Δ*SEN1990::frt* strain showed reduced weight loss and development of symptoms related to the disease compared to the WT strain (Figure 5D). Despite this, survival assays showed that the deletion of *SEN1990* did not improve the survival rate of the infected mice (Supplementary Figure 5). To better visualize all of the data gathered from the mice infected with the WT and Δ*SEN1990::frt* strains, we performed a principal component analysis (PCA), including mice weight reduction and clinical score, bacterial loads in every organ, and ileum and colon length. We observed that the mice infected with the mutant and the mice infected with WT formed distinct clusters, indicating that the strains appeared to behave differently in their pathogenicity (Figure 5E). Additionally, to begin the characterization of how the gene could reduce the colonization of sterile organs, we performed bacterial uptake assays by a murine macrophage cell line (RAW264.7). The cells were inoculated for 2 h with either WT or Δ*SEN1990::frt* expressing GFP ectopically through the pKK233.2-*gfp* plasmid and treated with gentamycin to kill the extracellular bacteria. In this assay, we did not find any differences in the bacterial internalization by macrophages between the strains (Supplementary Figure 6).

## Discussion

Despite the known involvement of ROD21 in the crossing of *Salmonella* ser. Enteritidis through the intestinal epithelium and the subsequent colonization of sterile organs (Pardo-Roa et al., 2019), little is known regarding the genes that this EGI harbors. Moreover, the fast advancement of computational protein modeling requires a constant revision of previously unknown gene products (Jeffery, 2023). Therefore, since only a few ROD21 genes have experimental or computational data regarding their function, in this study, we sought to gather current bioinformatic data to facilitate further characterization of its encoded proteins and their role in the bacterium pathogenicity. Among the results, we found new data on two uncharacterized lipoproteins. SEN1982 was found to contain a motif structurally similar to the wHTH domain of PadR transcriptional regulators. Moreover, *Salmonella* ser. Enteritidis strains lacking this gene had attenuated colonization of the spleen and liver in mice (Silva et al., 2012). Therefore, *SEN1982* might encode a DNA-binding lipoprotein that either regulates the expression of other virulence factors, functions as a toxin, or interacts with exogenous DNA (Kovacs-Simon et al., 2011; Kavanaugh et al., 2019). On the other hand, SEN1984 was found to be structurally similar to a β-barrel assembly component, BamE, which is a non-essential lipoprotein of the assembly complex that participates in the insertion of proteins in the outer membrane of Gram-negative bacteria (Knowles et al., 2011). Additionally, the RefSeq annotation for *SEN1994* and *SEN1995* indicated that these genes encoded a putative nucleotide sensing system possibly related to the formation of membrane pores, which was composed of a transmembrane protein with a SLATT-domain and a nucleotidyltransferase, respectively (Burroughs et al., 2015; Burroughs and Aravind, 2020). The absence of *SEN1994* resulted in the attenuation of *Salmonella* ser. Enteritidis colonization of the murine spleen and liver, while the absence of *SEN1995* attenuated the colonization of the spleen (Silva et al., 2012). Altogether, this suggested that ROD21 involvement in the capacity of *Salmonella* ser. Enteritidis to cross the epithelial barrier and colonize sterile organs could be due to the production of proteins that interact with the outer membrane of *Salmonella* ser. Enteritidis, the host cells, or neighboring bacteria.

On the other hand, SEN1990 was annotated through the RefSeq pipeline as a DNA-binding protein from the RepL family of Firmicute plasmid replication/maintenance proteins. We identified that SEN1990 shares structural similarities with transcriptional regulators from the wHTH superfamily. Because SEN1990 has been reported to affect the persistence of *Salmonella* ser. Enteritidis inside oviduct cells in chickens (Raspoet et al., 2014) through microarray-based screenings, we sought to further characterize this protein. We identified through *in silico* modeling that SEN1990 contains a three-stranded wHTH domain, supporting the notion that it is a DNA-binding protein (Aravind et al., 2005). In this type of wHTH, the additional β-strand interacts through hydrogen bonds with the basic residues in the C terminal strand of the domain (Aravind et al., 2005). In this model, the residues Asp-91 and His-127 are predicted to form a hydrogen bond. In the wing motif, His-127 was conserved among all SEN1990 homologs, whereas within the additional β-strand, Asp-91 was not conserved in all the homologs, but Asp-88 was, suggesting that this interaction could be rather between His-127 and Asp-88. Such a local deviation in the model generated could be expected even when the model has a TM-score above the threshold (hence a correct global topology) since this score was insensitive to local modeling errors.

Interestingly, we found homologs of SEN1990 encoded in other EARL EGIs that group in two distinct clusters based on their sequence similarities. The homologs from the first cluster were harbored by islands that were closely related by their integrase phylogeny to ROD21 and to plant pathogens, the latter being the most ancestral EARL EGIs known (Piña-Iturbe et al., 2018). On the other hand, the homologs of the second cluster were harbored by EGIs that were proposed to have originated more recently (Piña-Iturbe et al., 2018). Moreover, all the genes encoding the homologs were located in similar loci within their respective islands, which hinted a possible common function for these proteins. The conservation among EARLs suggested that SEN1990 homologs had an advantageous function for the islands or their host, albeit not essential since SEN1990 homologs were not ubiquitous among EARL EGIs. Further, the deletion of *SEN1990* in this study through lambda-red recombination was uncommonly difficult, which could be explained by the importance or inaccessibility of this locus in ROD21.

Since SEN1990 was predicted to belong to the RepL family of proteins involved in plasmid replication, we hypothesized that this protein could function in the replication/maintenance of ROD21, which was supported by the prediction of the DNA-binding wHTH domain. Despite this, the alignment of the amino acid sequences of SEN1990 and the proteins from the RepL family (Lampson and Parisi, 1986; Hefford et al., 1997) did not show strong similarities between the proteins. In addition to this, the alignment of SEN1990 homologs from other EARL EGIs showed several residues that were conserved even among the most dissimilar proteins but were absent from RepL proteins such as the region between Lys-118 and Leu-121. Furthermore, we demonstrated experimentally that the absence of *SEN1990* from the *Salmonella* ser. Enteritidis genome did not affect the extrachromosomal replication nor maintenance of ROD21. Nonetheless, ROD21 is known to transfer from its host to nearby bacteria through conjugation (Salazar-Echegarai et al., 2014), which in other EGIs involves the replication of both the remaining and transferred circular ssDNA (Johnson and Grossman, 2015). Therefore, it remains to be assessed whether *SEN1990* could be involved in the replication/maintenance of the dsDNA molecule after the island conjugation. It is worth noting that in this study, we also demonstrated that ROD21 is most likely found as a single copy per cell since the number of ROD21 copies in a bacterial population is approximately the same as the number of chromosomes. This suggested that ROD21 does not autonomously replicate as an extrachromosomal element but rather alongside the host chromosome while in its integrated stage. Nonetheless, since the amount of excised ROD21 was low compared to its integrated stage (3% of the total chromosomes), a subset of replicating ROD21 could remain undetected by the qPCR sensitivity and the natural variation of the samples. Therefore, it would be necessary to further assess the extrachromosomal stage of ROD21 through other means to confirm this finding. It is worth noting that a limitation of this work is that all structural analyses were performed *in silico*, which could deviate from the empirical structure of SEN1990. Therefore, to further characterize this protein and its homologs carried by EARL EGIs, its DNA-binding properties and structure must be determined *in vitro*.

While searching for the function of SEN1990, we found through the transcriptomic analyses of *Salmonella* ser. Enteritidis that the gene *oafB* encoded in the SPI-17 was downregulated in the mutant lacking *SEN1990* compared to the WT strain, and the ectopic complementation of *SEN1990* in the mutant strain recovered the expression of *oafB*. These data, together with the bioinformatic characterization of SEN1990, suggest that this gene could be encoding a transcriptional regulator. However, the capacity of the protein to bind to the promoter of *oafB* is yet to be demonstrated *in vitro*. Moreover, the expression of *SEN1990* in the complemented strain was significantly higher than the WT strain (Supplementary Figure 4A), but the expression of *oafB* was similar between them. This could be explained by errors in the protein function due to an overexpression (Supplementary Figure 4A), that the expression of *oafB* was limited by other factors, or that the SEN1990 did not regulate the expression of *oafB* directly. The SPI-17 *oafB* was previously known as family II *gtr*C, which is involved in the O-antigen rhamnose acetylation in *Salmonella* spp. (Davies et al., 2013; Kintz et al., 2017; Pearson et al., 2020). O-antigen acetylation in *Salmonella* has been shown to contribute to population heterogeneity, lead to bacteriophage resistance (Kintz et al., 2015), be essential to develop vaccine-mediated immunogenicity (Konadu et al., 1996), and contribute to the invasive capacity of iNTS-producing strains (Kintz et al., 2015). Additionally, *oafB* is preceded by *gtrA* and *gtrB*, which comprise an operon subject to phase variation controlled by OxyR and Dam (Broadbent et al., 2010). Despite their differential expression not being statistically significant, both *gtrA* and *gtrB* are also downregulated in the mutant strain. Particularly, *gtrA* also showed a downregulated expression like *oafB* through RT-qPCR (Supplementary Figure 4A), although one sample had an increased expression, suggesting that in some cases, other factors might activate the operon when SEN1990 is absent. Therefore, it would be interesting to evaluate if SEN1990 is involved in the phase-variation of the operon. Additionally, within the genome of the strains carrying EARL GIs with *SEN1990*, only *Salmonella enterica* serovars contained *oafB*, and other putative acetyltransferases with similarities to *oafB* were present in *E. coli* 0127:H6, *Yersinia intermedia* Y228, and *Serratia marcescens* UMH3. Since SEN1990 is highly conserved among most EARLs that encode it, this suggests that it is most probably not a single-target regulator. Moreover, since more ancestral EARLs compared to the ones found in *Salmonella enterica* contain *SEN1990* (Piña-Iturbe et al., 2018), the modulation of *oafB* could be a new function of the protein in this species.

The downregulation of *oafB* in the strain lacking *SEN1990* could be related to the diminished capacity of the bacterium to colonize organs (Silva et al., 2012; Raspoet et al., 2014) since impairments of the O-antigen modifications in *Salmonella* could diminish the LPS heterogeneity of the population (Avraham et al., 2015), leading to defects in the bacterium immune system evasion or changes in its interaction with the intestinal cells, although *oafB* does not significantly impact the human serum survival of its host pathogen (Kintz et al., 2017). However, we could not detect any change in the LPS structure through SDS-PAGE, which could mean that the downregulation of *oafB* by the absence of *SEN1990* was not sufficient to alter the LPS structure. Nonetheless, it is possible that the sensitivity of the technique did not allow for the detection of the O-antigen acetylation by *oafB* (Davies et al., 2013); thus, another more sensitive technique, such as mass spectrometry or antibody binding could be used to confirm this result. Moreover, it has been reported that the deletion of ROD21 and its impairment to excise from the *Salmonella* ser. Enteritidis chromosome upregulates the expression of an SPI-1 gene, *invA* (Pardo-Roa et al., 2019). Therefore, in addition to the regulation of *oafB* from the SPI-17 by *SEN1990*, this suggests a crosstalk between genomic islands and a putative new role of ROD21 in the modulation of the gene expression of these elements. Nonetheless, the evaluation of the gene transcription of the strain lacking *SEN1990* was only performed under normal laboratory conditions (LB broth at 37°C), which are not representative of the milieu to which the bacterium is subjected during an infection. Therefore, it could be possible that *SEN1990* modulates the expression of other genes in such environments, especially since it contributes to the effect of ROD21 on the bacterium pathogenicity. Hence, the next step would be to evaluate the transcriptome of the mutant strain under an infection-mimicking environment.

Interestingly, we found that when the strain lacking *SEN1990* was co-administered with the WT strain, there were no differences in the bacterial loads detected. On the other hand, when both strains were administered separately, the mutant strain showed a diminished capacity to colonize sterile organs, which suggests that the reduction of the bacterium pathogenicity due to the absence of *SEN1990* could be overcome by a cooperative effect between both strains, where the strain lacking *SEN1990* takes advantage of the WT strain (Diard et al., 2013).

When mice were infected with the strains marked with antibiotic resistance, the differences in bacterial load between the strains were more evident. In a previous study from our laboratory, we reported that there were no differences in the colonization of the spleen and liver between isogenic strains harboring either the *cat* or *aph* genes in an infection competition assay in mice (Tobar et al., 2013). Therefore, the differences between the strains were not due to an imbalance in the burden imposed on the pathogen by expressing antibiotic-resistance genes. This suggested that the reduced colonization that we observed in the sterile organs was a true effect of the absence of *SEN1990*. Moreover, the survival curves of mice infected with the strains with antibiotic resistance genes and without them showed 100% lethality, which suggested that SEN1990 does not impair the pathogenicity significantly. Nonetheless, the strains showed inconsistent results regarding the time when lethality started and how long it took to cause total lethality; hence, we could not draw conclusions regarding this. Regardless, we expect that the lethality would not be significantly impacted given that the deletion of the whole EGI only delays it by 24 h, and the total survival rate is 20% (Quiroz et al., 2011). Moreover, we did not observe differences in the colonization of the spleen in the strains without antibiotic resistance genes, indicating the colonization defects could be related to the blood and liver environments specifically. We also did not detect any differences in the internalization of the deletion mutant by murine RAW264.7 cells, suggesting the engulfment and later translocation of the pathogen by macrophages might not be affected. Altogether, we hypothesize that *SEN1990* slightly delays the development of bacteremia and colonization in other sterile organs, which is possibly related to the role of ROD21 in penetrating the intestinal epithelial barrier (Pardo-Roa et al., 2019). Whether this effect on the pathogenicity of the mutant strain is due to a downregulation of *oafB* is yet to be addressed.

In summary, we provided evidence that the previously uncharacterized gene from the excisable pathogenicity island ROD21, *SEN1990*, is a predicted DNA-binding protein. It contains a wHTH domain that regulates the transcription of *oafB* located in the SPI-17, suggesting a crosstalk between these horizontally acquired elements. We also established that it is involved in the pathogenicity of *Salmonella* ser. Enteritidis.

## Data availability statement

The datasets presented in this study can be found in online repositories. The names of the repository/repositories and accession number(s) can be found below: GEO database accession number: GSE235252.

## Ethics statement

The animal study was approved by Scientific Ethical Committee for Animal and Environmental Care from Pontificia Universidad Católica de Chile under the approval code: 210707009. The study was conducted in accordance with the local legislation and institutional requirements.

## Author contributions

GH-E, AP-I, and SB contributed to the conceptualization and design of the project. GH-E, AP-I, OV, IS, JS-A, PP-S, YM-B, EC, PR, VS, FB, SC-G, and AA executed the experiments for the data gathering. GH-E performed the data analysis and wrote the draft of the manuscript. SB, AK, and CS support experimental designs, reviewed and edited the manuscript and obtained the funding and managed the resources. All authors contributed to the article and approved the submitted version.
