# Supplementary material for: SEN1990 is a predicted winged helix-turn-helix protein involved in the pathogenicity of Salmonella enterica serovar Enteritidis and the expression of the gene oafB in the SPI-17
Source: Front Microbiol. 2023 Nov 3;14:1236458. doi: 10.3389/fmicb.2023.1236458 (PMC10655114; doi:10.3389/fmicb.2023.1236458)
Supplement: Supplementary file 2 [file Image_1.PDF]

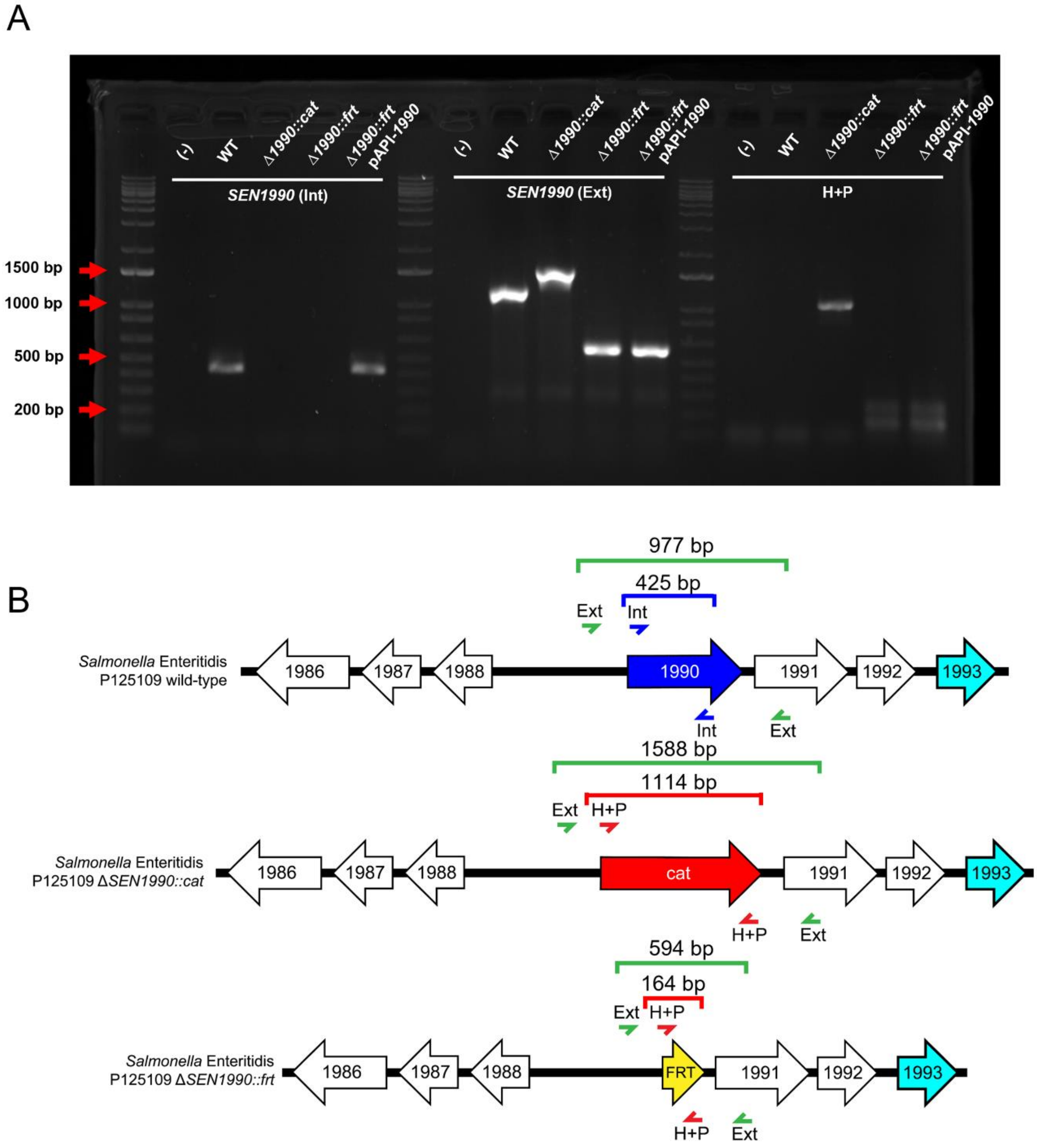

Supplementary Figure 1. Conventional PCR for the confirmation of the strains (upper half). **(A)** First half of a 1% agarose gel showing the PCR products for the confirmation of the strains. The primers used hybridize internally to SEN1990 (Int), externally to SEN1990 (Ext), and in the deletion scar sequence (H+P). The ladder used for size comparison of the amplicons was Invitrogen 1 Kb Plus DNA Ladder, and relevant sizes are displayed at the left of the gel. **(B)** Schematic representation of the primers hybridization in each strain.
