# Supplementary material for: SEN1990 is a predicted winged helix-turn-helix protein involved in the pathogenicity of Salmonella enterica serovar Enteritidis and the expression of the gene oafB in the SPI-17
Source: Front Microbiol. 2023 Nov 3;14:1236458. doi: 10.3389/fmicb.2023.1236458 (PMC10655114; doi:10.3389/fmicb.2023.1236458)
Supplement: Supplementary file 3 [file Image_2.PDF]

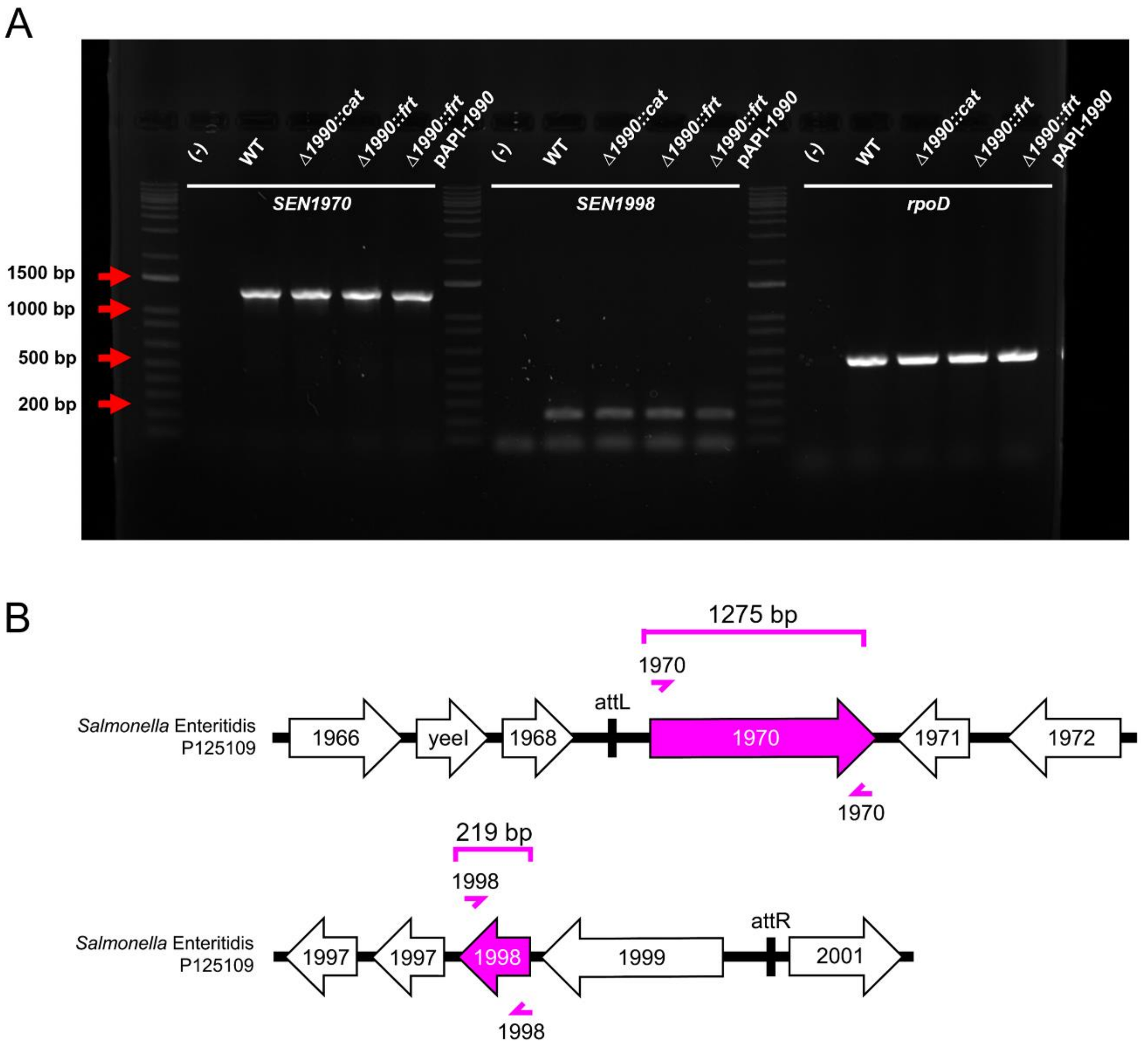

Supplementary Figure 2. Conventional PCR for the confirmation of the strains (Lower half). **(A)** Second half of a 1% agarose gel showing the PCR products for the confirmation of the strains. The primers hybridize internally to SEN197, SEN1998, and rpoD. The ladder used for size comparison of the amplicons was Invitrogen 1 Kb Plus DNA Ladder, and relevant sizes are displayed at the left of the gel. **(B)** Schematic representation of the primers hybridization in each strain.
