# Supplementary material for: SEN1990 is a predicted winged helix-turn-helix protein involved in the pathogenicity of Salmonella enterica serovar Enteritidis and the expression of the gene oafB in the SPI-17
Source: Front Microbiol. 2023 Nov 3;14:1236458. doi: 10.3389/fmicb.2023.1236458 (PMC10655114; doi:10.3389/fmicb.2023.1236458)
Supplement: Supplementary file 4 [file Image_3.pdf]

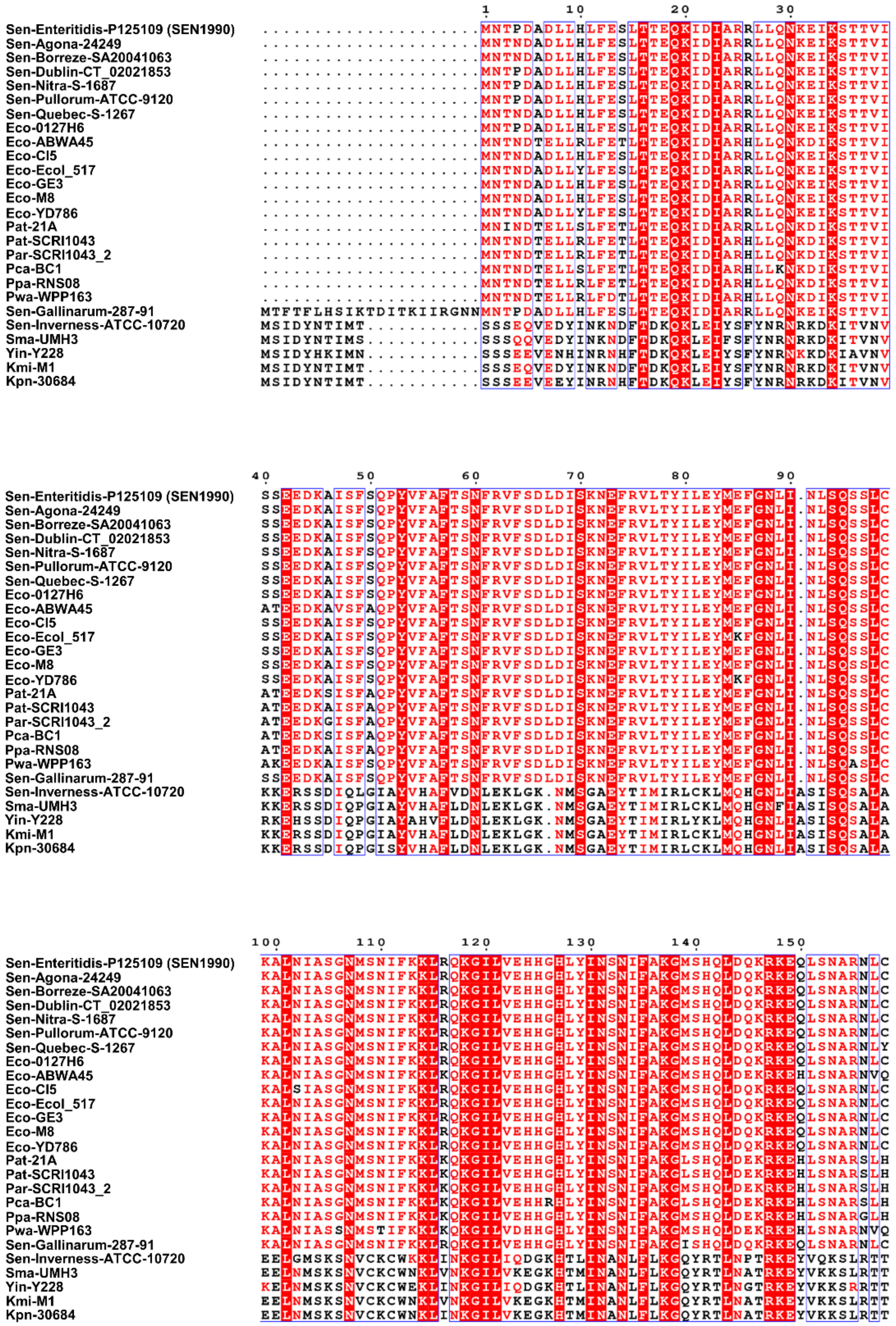

Supplementary Figure 3. Complete multiple sequence alignment between the SEN1990 homologs encoded in the EARL GIs. Residues highlighted in red columns are conserved in all proteins, while residues with red letters are highly conserved or share similar biochemical properties.
