# Supplementary material for: SEN1990 is a predicted winged helix-turn-helix protein involved in the pathogenicity of Salmonella enterica serovar Enteritidis and the expression of the gene oafB in the SPI-17
Source: Front Microbiol. 2023 Nov 3;14:1236458. doi: 10.3389/fmicb.2023.1236458 (PMC10655114; doi:10.3389/fmicb.2023.1236458)
Supplement: Supplementary file 5 [file Image_4.PDF]

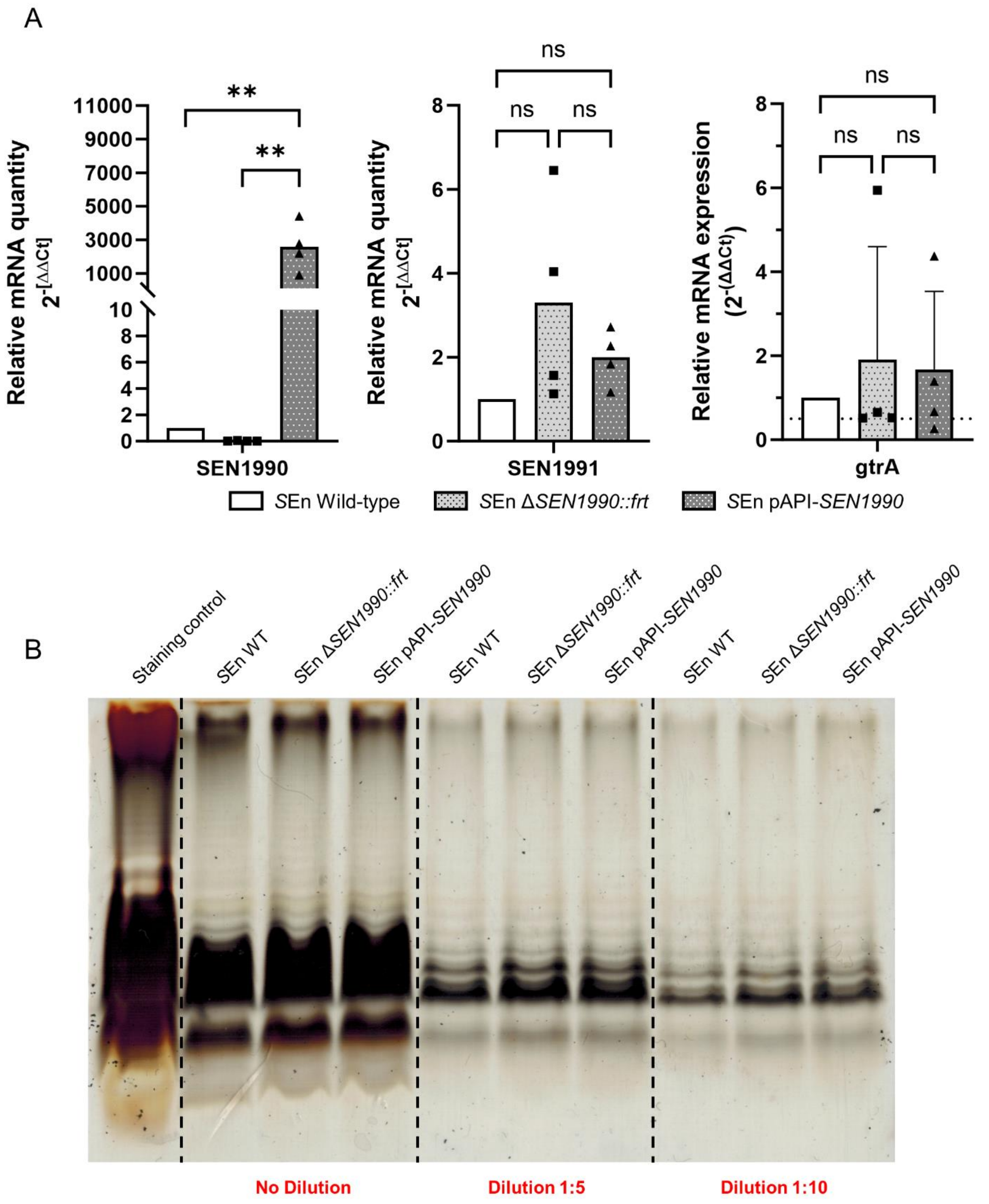

Supplementary Figure 4. (A) The relative mRNA quantity of *SEN1990*, *SEN1991* and *gtrA* between strains was calculated using the $2^{-(\Delta\Delta Ct)}$ method. The dotted line indicates the value 0.5. One way ANOVA for independent samples $\alpha = 0.05$. (** $P<0.01$). **(B)** SDS-PAGE gel with silver-staining of the LPS extracted from the WT, Δ*SEN1990::frt*, and Δ*SEN1990::frt* pAPI-*SEN1990*

strains. The first well shows a staining control from a previous LPS extraction from *Salmonella*. The samples are separated by a dashed line that indicates their dilution from the original.
