# Supplementary material for: SEN1990 is a predicted winged helix-turn-helix protein involved in the pathogenicity of Salmonella enterica serovar Enteritidis and the expression of the gene oafB in the SPI-17
Source: Front Microbiol. 2023 Nov 3;14:1236458. doi: 10.3389/fmicb.2023.1236458 (PMC10655114; doi:10.3389/fmicb.2023.1236458)
Supplement: Supplementary file 6 [file Image_5.PDF]

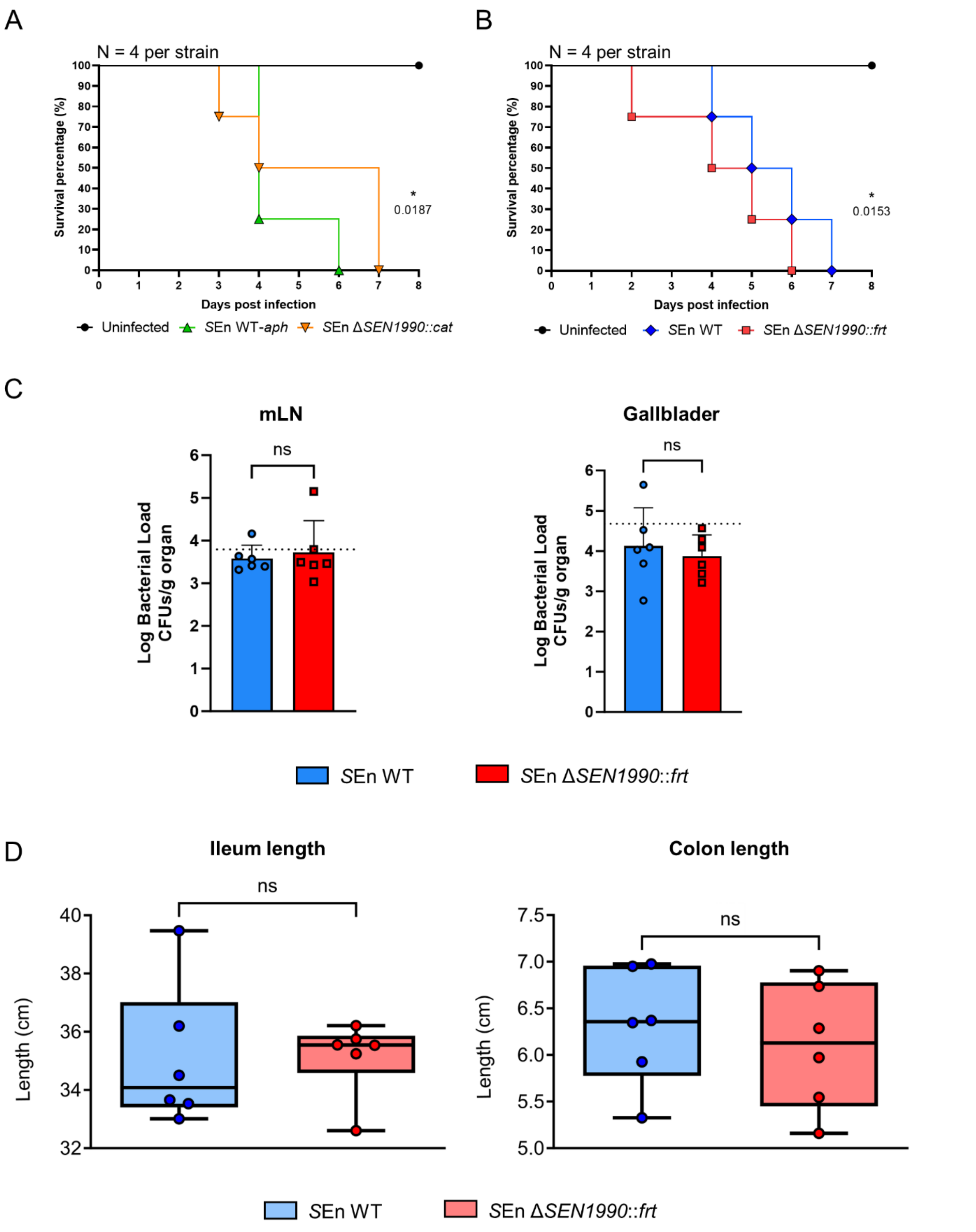

Supplementary Figure 5. Mice survival curves of the strains **(A)** with the antibiotic resistance gene and **(B)** without the antibiotic resistance gene. Each curve includes 4 mice for each strain. The curves were compared with the Log-rank (Mantel-Cox) test. (*P<0.05). **(C)** Log bacterial load

of the mLN and gallblader from the strains without antibiotic resistance. The dotted line indicates the LOD for each organ. T-test for independent samples $\alpha = 0.05$. **(D)** Length of the ileum and colon of the infected mice at 48 hpi. T-test for independent samples $\alpha = 0.05$.
