# Supplementary material for: SEN1990 is a predicted winged helix-turn-helix protein involved in the pathogenicity of Salmonella enterica serovar Enteritidis and the expression of the gene oafB in the SPI-17
Source: Front Microbiol. 2023 Nov 3;14:1236458. doi: 10.3389/fmicb.2023.1236458 (PMC10655114; doi:10.3389/fmicb.2023.1236458)
Supplement: Supplementary file 7 [file Image_6.PDF]

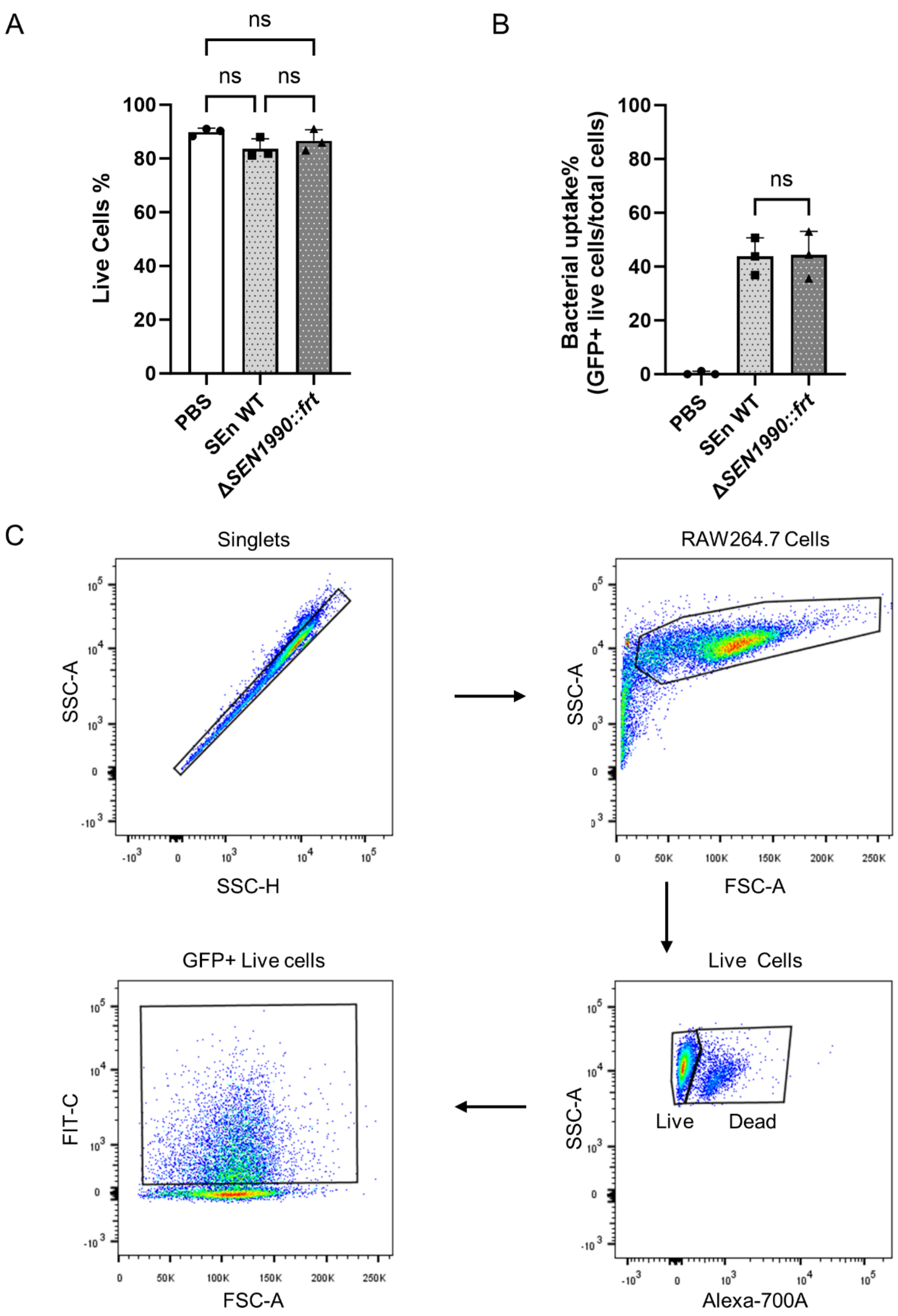

Supplementary Figure 6. Bacterial uptake assay by RAW264.7 macrophage cell line. **(A)** Viability of the macrophages after inoculation with the vehicle (PBS) or with the *Salmonella* ser. Enteritidis strains measured as cells negative for the viability probe signal. **(B)** Macrophages with

bacteria internalized measured as the percentage of live cells positive for the GFP signal by the total live cells in the sample. T-test for independent samples $\alpha = 0.05$. **(C)** Gating strategy example from one of the WT samples. Events are colored by the pseudo color scheme. Arrows indicate the flow of the gating strategy.
