# Supplementary material for: SEN1990 is a predicted winged helix-turn-helix protein involved in the pathogenicity of Salmonella enterica serovar Enteritidis and the expression of the gene oafB in the SPI-17
Source: Front Microbiol. 2023 Nov 3;14:1236458. doi: 10.3389/fmicb.2023.1236458 (PMC10655114; doi:10.3389/fmicb.2023.1236458)
Supplement: Supplementary file 8 [file Table_1.PDF]

Supplementary Table 1. PCR primers

Primers used to generate the strains

**Name**	**Sequence (5' → 3')**
SEN1990_H1+P1	TAAAACAGATATAACAAAAATAATAAGAGGGAA TAATATGGTGAAGGCTGGAGCTGCTTC
SEN1990_H2+P2	TGTTGTTGTTGTTATTTTATCAGGATACTTTCTG CATATGCATATGAATATCCTCCTTAG
pET15b _Fw	GGATCCGGCTGCTAACAAAG
pET15b_Rv	GCGTAGAGGATCGAGATCTT
ptrC_Fw	GGCGTAGAGGATCGAGATCTTATGGCTGTGCA GGTCGTAAATC
ptrC_Rv	GTGAGCGGATAACAATTTCACACCATATGAAAG TAAAAC
SEN1990-pAPI_Fw	ACACCATATGAAAGTAAAACTGGACTCTCTTAAC
SEN1990-pAPI_Rv	CTCATATGCAGAAAGTATCCTGAGGATCCGGCT GCTAACAAAG

Primers used to verify genotype

**Name**	**Sequence (5' → 3')**
SEN1990-Int_Fw	ATGAATACACCAGATGCTG
SEN1990-Int_Rv	TGATGACTCATTCCTTTAGCGAAA
SEN1990-Ext_Fw	GGACCAGTAAAATCGCGGCTTC
SEN1990-Ext_Rv	ATTTGAATGTGGCTACGGCACTG
SEN1970_Fw	TTGACGCATATGTCACTTACTGATACCAAAG
SEN1970_Rv	TATTCGGGATCCTTATTGTTTCTGAGCAAAC
SEN1998_Fw	TGAGCGCATATGGTTAAAAGAGAAATA AAAGC
SEN1998_Rv	TTATTTGGATCCTTATGCTGCGTTCTGG
rpoD_Fw	ATACACCAACCGTGGCTTGC
rpoD_Rv	TTCGCGGGTAACATCGAACT
