# Supplementary material for: SEN1990 is a predicted winged helix-turn-helix protein involved in the pathogenicity of Salmonella enterica serovar Enteritidis and the expression of the gene oafB in the SPI-17
Source: Front Microbiol. 2023 Nov 3;14:1236458. doi: 10.3389/fmicb.2023.1236458 (PMC10655114; doi:10.3389/fmicb.2023.1236458)
Supplement: Supplementary file 9 [file Table_2.PDF]

Supplementary Table 2. qPCR primers and probes

Primers and probes used to quantify gene expression

**Name**	**Sequence (5' → 3')**
SEN1990_Fw	GGAATACTCGTCGAGCATCATG
SEN1990_Rv	TGATGACTCATTCCTTTAGCGAAA
SEN1990-Probe	TCATCTCTATATCAACTCTAAC
SEN1991_Fw	TCTTGATTTTGTGGAGCGATCTT
SEN1991_Rv	GAAGGATTTCAAAAAGCAGGTTTG
oafB_Fw	ACTTGGCGCCTCTCTTATAA
oafB_Rv	CAACAGGCCTAACACTAAGC
rpoD-Fw	ACCCGGGAAGGCGAAA
rpoD-Rv	TGGTTGATCCCGTCTTCGAT
rpoD-Probe	CGACATCGCTAAACG

Primers and probes used to assess ROD21 replication, copy number and excision

**Name**	**Sequence (5' → 3')**
attB_Fw	GTTACTATGCGCCCCGTTCACAC
attB_Rv	CCGATTAAGCCCCAAAAACTATG
attB-Probe	TTCGAGTCCAGTCAGAGGA
rpoD-Fw	ACCCGGGAAGGCGAAA
rpoD-Rv	TGGTTGATCCCGTCTTCGAT
rpoD-Probe	CGACATCGCTAAACG
attP-Fw	AGCCACTAACAGCAGGAG
attP-Rv	CGAGAGTGAACCTGAAGAA
attP-Probe	TTCGAGTCCAGTCAGAGGA
