# Supplementary material for: SEN1990 is a predicted winged helix-turn-helix protein involved in the pathogenicity of Salmonella enterica serovar Enteritidis and the expression of the gene oafB in the SPI-17
Source: Front Microbiol. 2023 Nov 3;14:1236458. doi: 10.3389/fmicb.2023.1236458 (PMC10655114; doi:10.3389/fmicb.2023.1236458)
Supplement: Supplementary file 10 [file Table_3.PDF]

Supplementary Table 3. Mice clinical score parameters

Parameter	Criteria	Score	Periodicity
**Loss of body weight**	There is no loss of body weight, or it is less than 5%.	0	Daily
	Loss of body weight is between 5-10%.	1	
	Loss of body weight is between 10-20%.	2	
	Loss of body weight is higher than 20%.	3	
**Aspect**	Straight posture, hang from the cage, and its fur is lustrous.	0	Daily
	Avoids movement, fur is scruffy, might have red secretion from nose or eyes.	1	
	Curved posture, immobile and at the back of the cage, abulic, fur is scruffy, red secretion from nose or eyes.	2	
	Prostrate, lateral decubitus position, evident dehydration, sunken eyes.	3	
**Spontaneous behavior**	Aware of its surroundings, interacts with peers, grooms itself.	0	Daily
	Small changes: Reduces grooming and displacement, rapid respiratory movements, tends to remain at the end of the cage.	1	
	Wobbly displacement, inactive or separated at the back of the cage, abdominal breathing.	2	
	Mouse is immobile, lateral decubitus position, gaps mouth to breath.	3	
**Hydration**	Thoracic back skin return time is less than 2 seconds when pinched, normal posture, eye globe with no alterations.	0	Daily
	Thoracic back skin return time is less than 2 to 4 seconds when pinched, piloerection, body weight loss between 10 to 15%, reduced movement in the cage.	1	
	Thoracic back skin return time is between 2 to 4 seconds when pinched, body weight loss higher than 15%, piloerection, curved posture, weakness, and dry feces.	2	
	Thoracic back skin return time is higher than 5 seconds when pinched, curved posture, sunken eyes is evident, impossibility to stand by itself.	3	
**General evaluation of colitis**	Well-formed feces, no alterations of the anus	0	Daily
	Alteration of the feces consistency: Mushed or dry feces but well-formed, presence of mucus.	1	
	Soft feces or absence of depositions, hematochezia, slight alterations of the perianal zone such as edema, perianal dermatitis.	2	
	Diarrhea or constipation with tenesmus, hematochezia, might have melena, evident perianal alterations.	3	
